# Exploring patient perspectives on how they can and should be engaged in the development of artificial intelligence (AI) applications in health care

**DOI:** 10.1186/s12913-023-10098-2

**Published:** 2023-10-26

**Authors:** Samira Adus, Jillian Macklin, Andrew Pinto

**Affiliations:** 1 Faculty of Medicine, University of Toronto, Toronto, ON Canada; 2 Dalla Lana School of Public Health, Institute of Health Policy, Management, and Evaluation, Toronto, ON Canada; 3 MAP Centre for Urban Health Solutions, Unity Health Toronto, Toronto, ON Canada

**Keywords:** Patient engagement, Artificial intelligence, Qualitative research, Patient perspective, Application development

## Abstract

**Background:**

Artificial intelligence (AI) is a rapidly evolving field which will have implications on both individual patient care and the health care system. There are many benefits to the integration of AI into health care, such as predicting acute conditions and enhancing diagnostic capabilities. Despite these benefits potential harms include algorithmic bias, inadequate consent processes, and implications on the patient-provider relationship. One tool to address patients’ needs and prevent the negative implications of AI is through patient engagement. As it currently stands, patients have infrequently been involved in AI application development for patient care delivery. Furthermore, we are unaware of any frameworks or recommendations specifically addressing patient engagement within the field of AI in health care.

**Methods:**

We conducted four virtual focus groups with thirty patient participants to understand of how patients can and should be meaningfully engaged within the field of AI development in health care. Participants completed an educational module on the fundamentals of AI prior to participating in this study. Focus groups were analyzed using qualitative content analysis.

**Results:**

We found that participants in our study wanted to be engaged at the problem-identification stages using multiple methods such as surveys and interviews. Participants preferred that recruitment methodologies for patient engagement included both in-person and social media-based approaches with an emphasis on varying language modalities of recruitment to reflect diverse demographics. Patients prioritized the inclusion of underrepresented participant populations, longitudinal relationship building, accessibility, and interdisciplinary involvement of other stakeholders in AI development. We found that AI education is a critical step to enable meaningful patient engagement within this field. We have curated recommendations into a framework for the field to learn from and implement in future development.

**Conclusion:**

Given the novelty and speed at which AI innovation is progressing in health care, patient engagement should be the gold standard for application development. Our proposed recommendations seek to enable patient-centered AI application development in health care. Future research must be conducted to evaluate the effectiveness of patient engagement in AI application development to ensure that both AI application development and patient engagement are done rigorously, efficiently, and meaningfully.

**Supplementary Information:**

The online version contains supplementary material available at 10.1186/s12913-023-10098-2.

## Background

Artificial intelligence (AI) is a broad term referring to advanced computational methods that allow machines to mimic the functions of human cognition, such as learning or problem-solving [1]. AI is rapidly emerging as a technology that will impact numerous sectors, including health care. There is immense promise for AI to improve health care by surpassing the performance of health care providers, including assisting in the diagnosis of conditions such as melanoma and diabetic retinopathy [2, 3], predicting onset of acute conditions such as inpatient delirium or cardiac arrest [4, 5], and communicating with patients to address common questions as a chatbot [6, 7].

While AI has the potential to improve patient outcomes and health equity, potential harms exist. These include concerns about where patient data is being shared, the impact on the patient-provider therapeutic relationship, algorithmic bias, and the proper consultation of key stakeholders in AI development, among others [8]. Our ability to use AI in health has outpaced critical normative discussions among key stakeholders regarding how AI technologies should be responsibly developed and used in health care [9].

In Canada, the Canadian Institute for Health Research Strategy for Patient Outcomes Research defines patient engagement as “occurring when patients meaningfully and actively collaborate in the governance, priority setting, and conduct of research as well as in summarizing, distributing, sharing and applying its resulting knowledge” [10]. There is a growing consensus that patient engagement has a crucial role in health care delivery [11]. Effective patient engagement can improve patient outcomes, quality of life, and safety, as well as decrease hospital admissions and health care costs [12–14]. Engagement can also potentially lead to improvement in the acceptability of AI technology and support its transition into clinical practice [15]. Three in four patients feel that scientific developments should act in line with what is most important to patients and their families [16]. Patients are motivated to be involved in the development of new technologies as seen by our current understanding of the user-centered design space as being critical to developing digital health technology that users can and want to use [17]. Despite the goal of inclusive co-design, patients tend to still occupy passive roles in research through interviews or observations [18].

Specifically, within AI development, patient engagement has been largely overlooked. From a systematic review our research group is currently conducting, we found that very few studies on AI-related health care applications affecting patient care reported patient engagement in any form within its development. Additionally, of the patients who have been engaged, most identified as White, medically stable and have had a high enough educational attainment to have a fundamental understanding of AI [19]. We interpret these findings to be the product of a lack of a patient engagement framework or recommendations of how, when, and in what ways patients can be meaningfully engaged within this complex field. In doing so, we may work towards reaping the benefits of patient engagement within the field of AI and health care as a whole. Therefore, this articles aims to detail our findings and interpretations of how patient engagement can meaningfully be conducted within the field of AI, from the patient perspective, in addition to a preliminary framework for future patient engagement within this field.

## Methods

### Study design

This study utilizes an exploratory qualitative design using focus groups to engage patients on their perspectives of how patients can best be engaged in the development of AI in health care. We used the Sittig and Singh 2010 conceptual framework to guide our focus group question guide development [20]. As it currently stands, there are no conceptual frameworks addressing patient engagement research in AI, as such this paper will build towards the foundation of a conceptual framework to be utilized in future research.

### Study setting

This study was conducted virtually within Canada, and more specifically in the Greater Toronto area. Canada is a high-income country with a publicly financed single-payer universal health care system and diverse ethnicities. More than 85% of Canadians over age 12 have a primary care provider [21]. As of 2019, only 1.1% of Canadian physicians in any discipline reported using AI tools in patient care [22].

### Study participant eligibility and sampling

Participants were required to be 18 years of age or older, speak English, and have seen their health care provider in the last year. This definition included patients who have visited any type of provider, including nurse practitioners, social workers, physiotherapists, physicians and more. Given the setting of the study within the COVID-19 pandemic, participants required reliable devices and internet to participate in our virtual focus groups. Participants were on-boarded to the study via phone, consent was obtained verbally, and a demographic survey was administered. We employed the concept of maximum variation sampling – a technique otherwise used to identify dimensions of variation and selecting cases which fulfil this variation – to have diverse perspectives based on age, ethnicity, socioeconomic status, sex, gender, chronic illness, and geographical location across Canada.

### Recruitment

Participants were recruited from posters in Unity Health Toronto family medicine clinics, social media (Twitter and Kijiji), and through emailing various community organizations.

### Data collection

Given the novelty and complexity of AI, patient education prior to study participation was critical. Prior to participating in focus groups, participants were asked to complete a 30-min educational module on the fundamentals of AI, including the different types of AI, examples of its uses in clinical practice, and important ethical considerations. This module was created in joint efforts with an educator at the University of Toronto to review for readability. After the completion of this educational module, participants were asked to complete a feedback survey. The revised module has since been published on the Rise 360 platform and is open access for public use (see Additional File 1).

Semi-structured 90-min focus groups were conducted virtually on Zoom from July–September 2021. Our focus group question guide included questions about what stage of development patient engagement should occur, what barriers exist to engagement, and what tools or training are necessary for patients, among others (see Additional File 2). Fieldnotes were recorded and focus groups were audio-recorded and transcribed verbatim. Data was managed in Microsoft Excel. Data collection ended when thematic saturation was attained.

### Data analysis

Data collection and data analysis were performed simultaneously. This was a deductive qualitative analysis. We conducted a content analysis on all transcripts, where a team of coders (SA and JM) independently coded the same transcript manually using pre-conceived codes from the literature, and a codebook was developed. SA and JM both independently used the codebook in order to analyze the remaining transcripts line by line and each transcript analysis was combined across coders into main themes and subthemes. Member-checking with participants was performed as needed.

## Results

We have divided our results section into prominent areas of the patient engagement continuum, namely participant demographics, the need for patient engagement, patient recruitment, timing of engagement, engagement methods, patient education/training, the overall engagement process, and evaluation of the engagement. Please see Table 1 for representative participant quotes for each section.

**Table 1:** Representative participant quotes by engagement theme. NB: This table should be located in the Results section of this paper after: “Please see Table 1 for representative participant quotes for each section.”

Engagement Stage	Representative Participant Quotes
Need for Patient Engagement	*“In surveys, I also think that it's not uncommon for AI specifically, not to have a lot of patient engagement, because of the specific software and its use. So if it's diagnosing cancer and whatnot, patients wouldn't be using it, it would just be the doctor. So it's not surprising that they're not involved in that process.”* *“I think it's really important to have to expand the [AI] space and expand the people that are participating in it. Because with AI, as in a lot of things, it's the question of garbage in, garbage out. So if you have such a small sample [size], the information you’re basing your policies on will not be consistent with reality.”* *“Like, I personally, I'm not surprised, but it's very upsetting to see, you know, the majority of the participants were identified as white, because realistically, we cannot generalize that to the entire population. And it just shows that we need more race-based research focusing on black individuals, brown individuals, indigenous populations, and so on. Because at the end of the day, also, racial and ethnic minorities receive a lower quality of health care than white people. Like that's what all the studies say, right? And then that kind of ties back in with the patient engagement rollout, who are we actually reaching out to?”* *“And I'm just speaking out of the experience, as a patient advisor with a variety of health care settings, both in education and in hospitals, is that most of the patients participating, the patients really participating are usually very homogeneous. They have post-secondary education, they either have or have had work and are retired, they have a home, they have a certain level of income, they speak English, usually as their first language. And they're mostly white, mostly women, too. So mostly from a very, very homogeneous set of care. And it's been a struggle, because even if the organizations want to do more outreach to patients, the way the whole patient participation is set up, is sometimes not conducive to bring, for example, people who work all day and are only available in the evening, or on the weekends. Right. So there's the setup for participation also has a lot of barriers.”*
Patient Recruitment	*“I think that it should be a part of the clinics [where] every patient that comes in and is asked ‘hey, we need your input on this AI tech’. Because you need a diverse sample and people who are willingly going to want to participate in studies and focus groups they're definitely going to be similar in some way.”* *“And consider who is the population in Toronto, for example, there's a large majority of people whose language is not first language is not English. They we celebrate our diversity in the fact that we are in an immigrant city, that people learn English to work, they cannot read a paper. This is from my Latin American community, that's my experience, they don't know how to read or write. They don't want to rely on other people to actually take care of their health. So how are we making it fully accessible? Not only in terms of accessibility for persons with disability, which is the law, but also for those who are not health literate, who don't have the capacity to speak either official language?”*
Timing of Engagement	*“So to me, patient engagement in AI should invite the patient right at the beginning to even identify the problem: ‘what is the problem that we want to research?’ Because health care is a two sided coin, the priorities of the investigators and health care providers will be on how they can do the work best and more efficiently and more effectively for delivery. From the other side of the coin, from the patient perspective, I will be more interested in what are the barriers for me to actually access that health care, they give me the best medication prescription? Do I have the money for it? So I would like the question for research or the problem identified, to have input from people who are going to be the users.”*
Patient Education/Training	*“…having a round table with experts that are doing the actual AI, like a programmer, who does the algorithm…. Like having those high-end professions at the table as well as researchers. So if participants have a question, we get the answer right away.”* *“It’s up to the provider to educate, you should educate them on those things that they're not aware of, and it can take time. But that's what quality entails, you need to take more time to give good results, basically.”*
Methods of Engagement	*“There's not a lot of like qualitative perceptions. It's mostly through surveys of satisfaction and acceptability and AI interventions. And I think that if you want to engage the patient more, you need to talk to them.”* *“So I would think that a modular approach would be the best. And by modular I mean, invite a group for a three stage or three meetings, it could be one meeting in person, another meeting, online, *etc*. Just event, there's a variety of ways to participate. So you addressed a variety of communication styles and learning styles, you also have the opportunity to see familiar faces to feel that this is a safer space, I have not been harmed, I feel like impressed this group, in make it purposefully diverse.”* *“You could have small groups in libraries…libraries are a really good way to reach people and have computers so people who can't afford to have a phone or computer can also access it that way.”*
Process of Engagement	*“Also, I think that having funding for to say to the person, you know, if it is in person, we're going to give you lunch or dinner, we're going to pay for your transportation, we're gonna recognize your one hour commitment with this much money. So then persons may say, ‘yeah, I can skip my gig, because I can go to this place and still not lose income.”* *“So how are we making it fully accessible? Not only in terms of accessibility for persons with disability, which is the law, but also for those who are not health literate, who don't have the capacity to speak either official language? And so are we allowing ourselves a budget in timeframe to prepare the patient and inform the patient so then they can feel more appropriate? Because otherwise they just feel like oh, this is too educated for me this is too English for me and they self you know, self-eliminate before even participating”* *“But I also want to bring the concept of upstream. So, the researchers need to educate themselves, they need to also advocate with their schools, their faculties, because right now, today, all the researchers are graduating, are still being taught, from the perspective that health research is ‘how can we fix the problem?’ And when I show up at my doctor's office, I am the problem. And they want to fix me, not the disease, they want to fix me. And it's like, no, I have a chronic illness. It's never going to be fixed. So how about we both have a chat, and we learn how to manage the disease in a way that benefits my day to day living? So, they are still looking at patient equals disease equals deficit, and we need to flip that conversation. Because patients are empowered, I'm in charge of my life, not my doctor. So then how can my doctor help me with that? Right, so we need to go upstream, educate the researchers, but the researchers also need to look at their schools or an inclusive type of education.”* *“The second part to that, for that to be effective would be to recognize the patient's knowledge and experience as a skill. I said, my experience of disability is a skill. It took me 30 years to know everything I knew about my disease, I didn't choose this, but I have to have that knowledge. That's my skill. So when I participate in I bring that knowledge, I think it should be recognized because when I invite another professional that is going to bring their hard earned knowledge, it's usually recognized.”* *“I was invited to create a workshop for PhD and master students who were actually doing some research about diseases and treatments, and they have never, ever talked to a patient. So they were looking at the patient as an object, of ‘how can I fix this disease’, but they didn't know how that disease impacts the life of the person. Will the person be able to adopt and embrace your research solution? So I think the culture of inclusion of seeing the patient as a true partner in their health care, it's a learning curve.”* *“First, contact the general population, educate them, and then ask for the participation. So a few of those might want to participate when the research is done. So you go back to the patients who were engaged and say, ‘this is the publication, do you have anything else to add?’, if they have helped you write the paper, acknowledge them as co authors. Go back to the community and tell them listen, we did this research, this is how it's going to be published. And the next time around the community will embrace and say, yes, we want to participate, because we're seeing, they see us not just at the token, there's not an extractive process of our knowledge, but it's actually enriching it back, when they're finished, they bring back that knowledge.”* *“For everyone, the researchers, but also the patients that you engage, to educate them in an anti-oppression framework. So then, with my participation, I'm not just saying Ladies and gentlemen and maybe harming a person who was not binary in their identity. And that's extremely important that as a patient, my participation with all the love that I bring is not harming others in the process.”*
Evaluation of Engagement	*“I think it's a very difficult thing to do, because there's not a final product. So the engagement process is a continuum. So I would say that having the goal in mind and say, for this particular research, treated as a quality improvement project.”* *“I feel like it's almost impossible to know that up front, you'd have to like, incorporate the policies that were developed from the results, and then see if it improves health after a few years of actually implementing those policies.”* *“So different, like multiple kinds of opportunities to say and share what's happening with me as a patient. I think that's important.”* *“Other than an evaluation at the end of the project, to allow the patients for very honest and open feedback? Did they feel engaged? Did they feel that the knowledge of the subject in that particular project transformed? Like they have gained or learned something from it? And have they seen that the input, not just my input, but the input that the group was focusing more towards was actually considered?”* *“I guess a short answer: good quality patient engagement is: a) include the patient right from the start and b) make it fully accessible including time/funding to properly do it”*

### Participant demographics

We recruited 30 participants across 4 patient focus groups, ranging from 5–8 participants per focus group. Please find a detailed description of participant demographics in Table 2. In summary, 67% of our participants identified as female and the average patient age was 35 years old. We found that most participants identified as White, Black or South-Asian. Most participants resided in the Greater Toronto Area in Ontario, with a couple of participants from British Columbia. Most participants have completed college/university level degrees and self-identify as being moderately knowledgeable about AI. None of our participants expressed having worked within the AI field prior to attending the focus group. Approximately a third of participants self-identified as experiencing chronic illness and a third of participants expressed having accessibility needs.

**Table 2:** Participant Demographic Data

Participants	
**Characteristic**	**# (%) of Participants (*N* = 30)**
**Age, mean (range)**	35 (18–73)
**Gender**	
Male	10 (33)
Female	20 (67)
Trans Female/Trans Woman	0 (0)
Trans Male/Trans Man	0 (0)
Two-Spirit	0 (0)
**Race/Ethnocultural group**	
Asian – South (India, Pakistan, Sri Lanka)	8 (27)
White/European/North American	6 (20)
Black – Africa and Caribbean	6 (20)
Asian – East (China, Japan, Korea)	3 (10)
Asian – Southeast (Vietnam, Philippines, Malaysia)	2 (7)
Indigenous (Inuit, Metis, First Nations)	0 (0)
Hispanic/Latin	1 (3)
Middle Eastern (Iran, Egypt, Lebanon)	3 (10)
Prefer not to answer	1 (3)
**Difficulties making ends meet at the end of the month?**	
Yes	7 (23)
No	23 (76)
**Highest level of education**	
Some grade school	0 (0)
Some high school	0 (0)
High school	1 (3)
Some college/university	8 (27)
College/university	21 (70)
Post-graduate	0 (0)
**Perceived AI knowledge**	
Not knowledgeable at all	2 (7)
Slightly knowledgeable	7 (23)
Moderately knowledgeable	16 (53)
Very knowledgeable	3 (10)
Extremely knowledgeable	2 (7)
**Currently experiencing chronic illness**	
Yes	8 (27)
No	13 (43)
Prefer not to answer	9 (30)
**Self-identified accessibility needs**	
Yes	8(27)
No	13 (43)
Prefer not to answer	9 (30)

### The need for patient engagement

To start the focus group discussion, participants were presented with recent systematic review findings on the prevalence of patient engagement in AI development in health care. From these findings, participants expressed a mixture of surprise and anticipation. Some participants described surprise that although patient engagement is well-known to be beneficial, we are still so far off from doing it well in the AI space. Other participants were not surprised, yet still upset, by the lack of patient voice. Nonetheless, the majority of participants stated patient engagement is critical for inclusion in AI development processes, while highlighting their expectation of patient engagement being a new standard in all AI development, as with any other field.

A common theme when discussing the need for patient engagement was the importance of diverse patient representation across social determinants of health and background (e.g., low income, racialized, English as second language, etc.) for those who are engaged, and that the lack thereof thus far contributes to health inequities and low generalizability. One participant stated: *“Because with AI, as in a lot of things, it’s the question of garbage in-garbage out. So if you have such a small sample [of patients engaged], the information you’re basing your policies on will not be consistent with reality.”* Another participant stated: *Because at the end of the day, racial and ethnic minorities receive a lower quality of health care than White people. Like that’s what all the studies say, right? And that kind of ties back in with patient engagement roll-out, who are we actually reaching out to?”.*

A secondary theme that emerged was the idea that if the AI technology is meant to serve the physician in doing their tasks, such as a diagnostic tool, then perhaps patients do not need to be engaged in those applications. However, it was also mentioned that physicians should serve as the bridge between the AI technology and patients, as they are still by proxy end-users.

When discussing the need for patient engagement in AI applications, concerns with respect to AI integration in health care were expressed. Specifically, the removal of the humanistic component of medicine, fears of data privacy and storage, the lack of consenting processes and patient notification pathways, and the worsening of health inequities through biased algorithmic design/data. However, many participants highlighted patient engagement as being a method of addressing patient and community needs in addition to it being used as a tool to foster acceptability of AI interventions. One participant highlighted this here: *“I think that good patient engagement in general can help build trust, I guess, with the health care providers and just with the health care system itself. So I feel like when you're introducing something new, such as AI, people are kind of more willing to, if not accept then even just listen and kind of understand what's going on.”*

### Recruitment

Participants discussed two key components of where, how and who to recruit for engagement in AI application development. A recurring theme was performing recruitment in primary care clinics, rather than hospitals, as a method of engaging a large representative group of patients in addition to leveraging primary care physicians’ longitudinal relationships with their patients. Another major theme was the need for recruitment in spaces where racialized populations are located geographically, and through community organizations that patients trust, such as churches or neighborhood community centres. In order to engage intergenerational perspectives, some suggested the need for recruitment in long-term care homes to engage older adults.

When discussing how to recruit engaged patients, participants placed emphasis on having multiple recruitment avenues, including information boots in clinics and hospitals to have in-person recruitment, as well as using social media specifically to recruit younger generations and those digitally connected. The majority of participants, particularly living in Toronto, urged recruitment materials to be translated to commonly spoken languages to ensure that researchers are not excluding non-English speakers. It was particularly important in the field of AI, as this field uses complex language and terminology that patients with English proficiency as a second language may still have difficulty interpreting.

In terms of who should be engaged, participants emphasized the need for both patients and their caregivers to be recruited. Further, some participants highlighted the need for interdisciplinary collaboration with simultaneous recruitment of developers, programmers, researchers, physicians and policymakers.

### Timing of engagement

There was a resounding emphasis on the need for patient engagement from the very beginning of AI development at problem identification and prioritization stages. As one participant stated: *“So to me, patient engagement in AI should invite the patient right at the beginning to even identify the problem: ‘what is the problem that we want to research?’ Because health care is a two sided coin, the priorities of the investigators and health care providers will be on how they can do the work best and more efficiently and more effectively for delivery. From the other side of the coin, from the patient perspective, I will be more interested in what are the barriers for me to actually access that health care or technology.”* Some participants also highlighted that early engagement would provide the opportunity to save technological resources and prioritize future developmental iterations appropriately. A smaller minority of participants noted that the timing of engagement may be dependent on the end-user of the intervention itself, and in instances where physicians are end-users, prioritizing physician engagement during these stages and during later stages of development consulting patients. Importantly, the majority of participants agreed that providing choice to patients in terms of which stages and to what degree they would like to be engaged in the development process is essential, as every patient has a different agenda, ability, and interest.

### Patient education and training

Participants highlighted AI education as being a critical component to patient engagement within this field so that they may meaningfully engage. Although some participants noted that patients do not need to know everything about AI, researchers and AI developers should determine which level of basic understanding is fundamental for quality patient contribution.

Few participants reported having worked or learned about AI prior to completing our patient educational module. As such, the novelty and complexities of AI pose a challenge and may have implications on participation. It was a common participant worry that highly educated patients with higher income would be more involved in AI technology than those with less education and lower income, creating a class divide in patient engagement. Another concern was that older patients may be hesitant to participate if they are not comfortable or savvy with technology. Importantly, one participant mentioned that having patients with very little AI knowledge to start is important, as it is representative of the general public.

On the topic of whose responsibility it is to educate patients on AI, one participant highlighted the role of physicians as being direct patient educators. It was also suggested to have interdisciplinary experts involved in future patient engagement team training, in order to answer patients questions about AI and further their understanding in-person, in real time. Importantly, a common theme was that patient education takes time, but “*that is what quality entails, and we must take the time and energy necessary.”*

Participants enjoyed our educational module and appreciated the learning. From our feedback received on the module, we found that it took participants 25–35 min on average to complete the module, with a global rating of there being slightly too much content. The most challenging reported sections were those on AI methodologies, namely machine learning, natural language processing, and deep learning. In contrast, users generally found the ethics section the easiest. As a whole, participants rated the difficulty of the module as neither too easy nor too difficult. Areas highlighted for future improvement include the addition of videos and enhanced case studies. In terms of strengths, participants appreciated the use of images, glossaries, and real-life examples of AI. We found that age nor educational attainment impacted participants' self-rating of AI knowledge prior to completing our educational module.

### Methods of engagement

Participants emphasized the need for starting the patient engagement process with patient partners in mind, and choosing and creating engagement methodologies based on patient needs and preferences, while balancing feasibility concerns. It was well agreed upon that regardless of the situation, there should be a core group of patients engaged in a project from start to finish with a series of longitudinal meetings and continuity at each step to gain honest feedback and develop trust amongst patient partners. Other engaged patients may be involved in specific steps of the project, such as testing out an AI prototype.

Having a variety of engagement modalities was found to be an important topic in the focus groups, with some believing that both surveys and focus groups should be implemented as ways for patients to engage with AI applications. Participants often discussed the pros and cons to focus group and survey methodologies, specifically as it concerned sample size. Some patients expressed concerns with capturing a breadth of patient perspectives and experiences through focus groups, while others favored the quality of data to be had through focus groups in contrast to surveys: *“There's not a lot of like qualitative perceptions in patient engagement in AI. It's mostly through surveys of satisfaction and acceptability and AI interventions. And I think that if you want to engage the patient more, you need to talk to them [more in depth].* Others discussed that the method of engagement is contingent on the type of AI application itself, with some mentioning that focus groups may be more appropriate in the setting of trialing the intervention/product.

For the location of patient engagement, participants frequently mentioned the need for both on-line and in-person avenues for engagement. Mentioned in-person locations included sites like community centers and libraries that are easily accessible for patients, specifically as it pertains to patients without access to electronic devices. The majority of participants believed a mix of online and in-person meetings allowed teams to address a variety of communication and learning styles, and provide opportunities to have a familiar, in-person place that feels safe and comfortable for engaged patients.

For knowledge dissemination of patient engagement results, a similar emphasis on a multi-method approach was proposed. Some participants proposed researchers and AI developers send on-going updates of study progress and the usage of summary documents to be sent to all patient partners and study participants. Other participants found that town halls may assist in being able to engage not only the study partners and participants, but the larger community as a whole. Similar to patient engagement recruitment, participants suggested a mixture of formal (email) and informal (social media) pathways for knowledge dissemination, with the emphasis on accessibility and language translation, as needed.

### Process of engagement

We define the “process of engagement” as enablers for satisfactory patient engagement experiences. Participants discussed these enablers in three categories: patient-specific principles, provider-specific principles, and combined patient-provider principles. “Providers” include clinicians, researchers, and others on the AI application team.

### Patient-specific principles

For patients, important components for satisfactory patient engagement experiences were compensation, attentiveness to competing patient commitments, and accessibility.

For compensation, participants discussed the importance of AI teams allocating sufficient funds from the start for their patient partners and participants. Specifically, funds that cover potential lost wages and transportation costs, as well as funds for their participation time and energy. Providing a meal at team meetings was another form of compensation. Additionally, participants found it important that researchers are mindful of the other commitments patients may have with respect to their work or personal lives, and how this may affect their capacity to participate in engagement.

Another common topic of discussion among the focus groups was the importance of accessibility throughout the patient engagement process. Specifically, ensuring that different mediums of engagement have factored in the accessibility needs of participants both from the perspective of patients with a physical and/or mental disability, and from a health literacy perspective. It is important for AI teams to budget in time and funds “*to ensure patients do not self-eliminate before even participating.”*

### Provider-specific principles

Participants discussed important provider-specific enablers for improving the patient engagement experience. First, provider education was highlighted as an area for continuous development, specifically so that clinicians, researchers, and AI developers are educated on more upstream methods of engagement to garner representative patient sampling and the incorporation of diverse perspectives and experiences, as well as being educated on what meaningful engagement looks like. This was followed by a common theme of providers understanding how to develop and nurture community partnerships; not only looking to patients, but also to communities to assist in research problem identification, recruitment, and knowledge dissemination. Community engagement was highlighted as a way to gain trust with end users of the AI application, especially in communities that are notably marginalized or at risk of harm of AI applications. They discussed how community members who were engaged in the project also bring back a unique skillset and knowledge base that they can share with their community.

Second, many participants expressed the importance of researchers validating their patient knowledge as a skill, particularly in the environment of developing technology which seeks out to improve their lived experience of illness. “*My experience of disability is a skill. It took me 30 years to know everything I knew about my disease, I didn’t choose this, but I have to have that knowledge. That’s my skill. So when I participate in I bring that knowledge, I think it should be recognized because when I invite another professional that is going to bring their hard earned knowledge, it’s usually recognized.”* Another participant stated: “Some researchers *have never, ever talked to a patient. So they were looking at the patient as an object, of ‘how can I fix this disease’, but they didn't know how that disease impacts the life of the person. Will the person be able to adopt and embrace your research solution? So I think the culture of inclusion of seeing the patient as a true partner in their health care, it's a learning curve.”.*

Additionally, participants discussed the importance of adequate acknowledgment of contribution to the AI project of patient partners, not only in financial compensation, but also in academic authorship or recognition in reports and presentations. Participants all agreed that there was no room for tokenistic engagement where patients were included as a checkmark.

### Combined patient-provider principles

Participants highlighted that empathy and active listening were critical for patients and providers to work together in the engagement. Both patients and providers alike were discussed in terms of their importance in the engagement process, with providers initiating these opportunities with patients, and patients seeking out these opportunities themselves, as well. Throughout the patient engagement process, participants discussed the importance of decision-making power, such that patients being engaged feel and believe that they are able to enact change in AI development through the proposed engagement pathways. Anti-oppression frameworks were reported.

### Evaluation of patient engagement

Participants discussed how the topic of patient engagement evaluation is challenging, given the nature of patient engagement being an improvement continuum of long-term patient health outcomes and there may not be a final product in all cases. While some participants stated evaluation should come well after the implementation of the AI application, it was important to the majority of participants that there be incorporation of patient feedback at multiple time points throughout the longitudinal project, and not just at the end.

It was also discussed that the term “successful engagement” is difficult to define, because success will look different to different patients and teams. Importantly, participants reflected on the idea that effective patient engagement can be achieved only when the values of the project align with those of the participants, namely as it concerns research transparency and authenticity throughout the process. On the matter of markers of effective patient engagement, one participant suggested using the patient's sentiments of inclusion, adequate knowledge to participate, and a sense of self-improvement and gain. Specifically, if they feel like they are being engaged well, if they feel prepared to engage and if they have learned/gained anything throughout the process. For engagement of community organizations, it was also suggested to seek feedback from these organizations to foster a long-term relationship of engagement and trust.

## Discussion

**Fig. 1:**
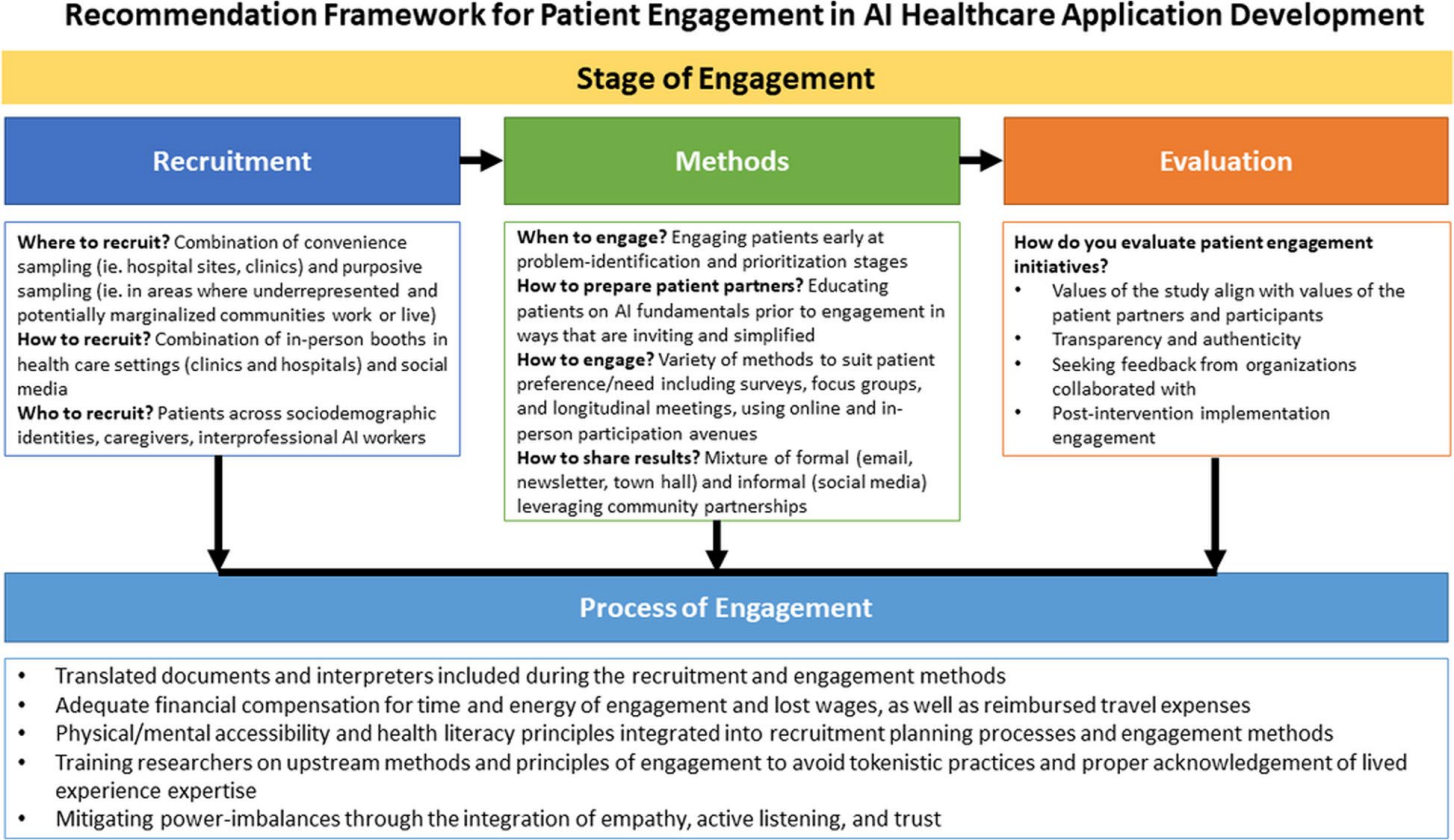
Recommendation framework for patient engagement in AI healthcare application development

Over the last decade, AI has demonstrated that it has a powerful role in its abilities to innovate the medical field. While there has been innovation from a technical standpoint in the field of AI in medicine, there has yet to be a formalized series of recommendations made for how patient engagement can be meaningfully done throughout the AI development process. Through a series of patient focus groups, we have begun to develop recommendations and a conceptual framework for how patient engagement should be conducted within AI application development in health care. Please see Fig. 1 for a summary of our recommendations.

Some of the most prominent themes from our discussions with patients were: the need for patient engagement, education on AI, interdisciplinary collaboration in AI as it pertains to patient engagement, equity diversity and inclusion (EDI), and quality improvement in patient engagement.

### The need for patient engagement in AI

It has been well-established that patient engagement is needed in health care, and the results of this study reiterate that it is similarly important in the field of AI in health care. Patient engagement, when done meaningfully, has the opportunity to ensure development is in alignment with patient needs, provides insight into reassuring patients concerns with respect to AI development, and create longstanding collaborative relationships between health care providers and the patients they serve.

AI development can be described as a life cycle comprising various stages from conception to production and implementation. One such model of this cycle is that developed by DeSilva and Alahakoon (2022) which discusses the CDAC AI life cycle comprising 3 phases: design, develop and deploy. The first stage within the design phase is problem identification [23]. Based on our data, we found that patients would prefer to be engaged at problem identification stages as a means of prioritizing and strategizing their own health needs. Participants within our study highlighted that there are many benefits to encouraging patient collaboration at problem identification stages including improvements to resource allocation. By partnering with patients in early stages, there may be benefits to avoiding future iterations of improvements due to latent patient feedback.

In addition to patient engagement being beneficial from an operational perspective, there are also benefits from an AI acceptability standpoint by both informing patients of technological advancements and being able to adequately address patients concerns through engagement [22, 23]. In a study investigating AI-led chatbot services in health care, researchers found that the employment of user-centered approaches to address patient concerns assisted in improving both user experience and utilization [24]. Within our study, patients discussed concerns of AI use, specifically as it pertains to data consent processes, representation of data and algorithmic justice, data privacy and storage ethics. These concerns have similarly been discussed in the literature [25]. To address these reservations, collaborative study design principles such as user-centered design and patient engagement can be used. It has been found that health and technology literacy contributes to people's perceptions of AI, and assists in building trust, further re-iterating the critical role AI education has in the ways in which patients interact with and adopt AI [26].

Patient engagement work should serve as a condition that needs to be met by AI researchers rather than an after-thought as it relates to the AI cycle of development.

### Patient education in AI

During our study, we drew importance on educating our participants about AI prior to entering focus group discussions. We created an introductory AI educational module which discusses the fundamentals of AI.

A main take-away from this work was that AI is a new, rapidly-evolving, and complex field that often makes patients feel unprepared, uncomfortable, and uneducated, oftentimes not even trying to engage in such a complex topic. It has been proposed in the literature that in order to successfully engage patients, there must be patient orientation and education about the topic, and on-going support [27]. This statement may be that much more important with the steep AI learning curve, which may necessitate more rigorous and longitudinal training throughout the engagement process, with the creation of accessible and easily understood educational modalities, than engagement studies may speak on their own lived experiences of a disease, for example.

As it currently stands within the literature, there are many papers discussing the importance of educating health care providers about AI in medicine from the perspective of digital literacy, specifically as it concerns medical students, physicians and nurses; however, none of which discuss educational pathways for patients themselves [28–34]. While educating health care providers is critical from an AI stewardship perspective, the lack of accessible learning modalities for patients re-iterates paternalistic structures in medicine whereby health care providers are the holders of knowledge. This directly contrasts the general principles of patient engagement as listed by the Ontario Patient Engagement Framework, which serves to empower patients and permit self-advocacy [35]. We hope that with the creation of our module we may begin to create more accessible educational pathways for patients and the general public to learn more about AI.

### Interdisciplinary collaboration in AI and patient engagement

The nature of AI development in health care is complex. There are a multitude of stakeholders within the field of AI including developers, data managers, clinicians, and ethicists, among others. In order to successfully research, develop and implement beneficial AI interventions, there must be interprofessional collaboration across these groups. As it currently stands however, there are no established strategies for interprofessional collaboration within the field of AI [36].

Within our discussions, participants acknowledged the importance of interdisciplinary collaboration and its potential integration in patient engagement methods by having experts answer patients' questions and provide contextual insight throughout the engagement process. While this may provide added benefit from an educational standpoint, and may assist in clarifying key concepts for patients, doing so may result in the creation of power dynamics. As such, if this method is to be adopted, these power dynamics must be mitigated. Data is power, and from which lends itself to being analyzed through decolonizing lenses of mitigating power-dynamics. One such indigenous research framework which both addresses and overcomes power dynamics of western research methodologies are Talking Circles. The purpose of Talking Circles is to build relationships across members of the Circle, share power, elicit stakeholder voice, sharing of ideas to solve problems and assist in shared design. The Circle method itself entails the researcher creating a safe space for participants to express viewpoints. This method places emphasis on the physical and spatial orientation of participants as equals, in addition to dedicating time to acknowledge individual participants' power and privilege in relation to the topic being discussed [36, 37]. As mentioned by Brown and Di Lallo, the Circle has potential to be used to mitigate power imbalances between participants and researchers, and among participants themselves [37]. General principles of talking circles can be applied within the context of overcoming power-dynamics within interdisciplinary collaboration work in AI, particularly those involving patients.

### Patient engagement in AI within an EDI lens

With respect to our recent systematic review demonstrating the lack of patient engagement specifically within marginalized communities, building longitudinal patient engagement relationships with members of marginalized communities is imperative. The inclusion of the voices of patients experiencing marginalization may serve as a method to combat the well-known implications that AI may have in worsening health inequities in health care, specifically with respect to a lack of represented in the data, and a lack of prioritization of anti-oppressive practices by AI researchers and developers [38]. A paper by Leslie et al. details the cascading effects of health inequities as they present in AI system development, namely the usage and perpetuation of discriminatory data and sampling bias, biased design and deployment, unethical applications of these biased models and the real world implications on health outcomes [39]. Often within our study we found participants referencing the earlier aspects of the cascade, with discriminatory data sampling and representation within the samples themselves and the broader picture of how this may impact their health. Additionally, participants discussed the importance of centering the patient’s experiences in patient engagement work as a skill to be valued, and in prioritizing the contributions of patients experiencing marginalization, AI engagement can be done meaningfully [40]. The notion of employing recruitment practices that purposely sample patients from marginalized communities is important; however, it is critical to avoid tokenistic practices when doing so [15]. In order to achieve this, research groups may instead collaborate with the communities they wish to engage in longitudinal relationships, with transparency and accountability to garner trust and improve patient engagement uptake. Building long-standing reciprocal relationships between researchers and patients being engaged can assist in fostering mutual respect, creating expectations and further informing future research priorities [41]. These research partnerships can also assist in mitigating language, socioeconomic, and cultural barriers which otherwise may impede patient participation in engagement. However, relationship building must be preceded by training from researchers themselves on anti-oppression and cultural humility [42].

### Quality improvement in patient engagement practices within artificial intelligence

The topic of quality improvement in patient engagement practices is an area of limited research both in terms of what to evaluate and how to do so [27, 40, 43, 44]. Currently, there are notable gaps in research assessing patient engagement, which may be attributable to the delayed impact patient engagement has on an individual and systems level, in addition to a lack of an agreed upon evaluation framework [40]. Furthermore, there is little research measuring the validity of indicators currently used for patient engagement. A study conducted by Vat et al. suggested the use of a coherent set of measures for effective patient engagement rather than a single measure such as recruitment rate [40].

In our study, patient participants described the importance of concordance of researcher and patient values as it concerns patient engagement research, specifically with respect to transparency, and trust as being important features of meaningful patient engagement. This finding has been supported by previous research suggesting 9 principles of quality improvement in patient engagement work, specifically discussing transparency, integrity, respect, and continuous re-evaluation [16]. From our study, subjective and objective measures of determining patient engagement success were outlined. Subjectively, participants outlined the use of surveys, either at the end of the engagement process, or as a continuum throughout the engagement process. Using these surveys, key areas of inquiry include the patient's subjective level of participation, preparedness for participation, and if they have in any way benefited from their participation. From this, we can understand that it is important that patients feel competent and heard when participating. Also emphasizing the importance of reciprocity in patient engagement, where the patients themselves can benefit from the process. Additionally, patients highlighted that a marker of quality patient engagement may rest in the researcher’s perceptions of the quality of the data itself. Objectively speaking, patients described that quality patient engagement may be measured by comparing the representation of social determinants of health within the sample of surveyed participants relative to the population as a whole. These principles can further reiterate and inform current models of patient engagement evaluation more broadly such as the Public and Patient Engagement Evaluation tool which includes a socio-demographic survey, an evaluation of communication and supports for participation, ability to express views while engaging, and perceived level of input/influence in the patient engagement initiative [43].

While patient engagement is critical for health care innovation, it is also important to acknowledge the time and financial resources required for its success which may cause tensions with research or clinical teams. Developing a better understanding of the markers for good patient engagement can assist in making the case to researchers and other stakeholders of its importance [40, 45, 46]. Given the significant investment and corresponding speed at which the field of AI is developing, it is critical to ensure that the implemented patient engagement practices are continuing to be evaluated. We argue that in this setting, evaluation is just if not as important as the methods of engagement themselves to ensure that patients are being appropriately consulted and that researchers are held accountable.

### Strengths, limitations, and future work in patient engagement in artificial intelligence

This study was a Greater Toronto Area-based study that emphasized the re-centering of the patient voice in artificial intelligence innovation in health care. Our study comes with several strengths. Our educational module for patient participants prior to the focus groups took an extra step in engaging patients to ensure they could contribute to thoughtful discussion. This module can now be freely used by other research teams and the public. We conducted an open-ended focus group approach where we primarily empowered patients to guide the discussion to topics that were important to them. We engaged a diverse group of patient participants based on age, socioeconomic status, and race, as well as various personal experiences with disability and chronic illness which may provide differing opinions on AI applications in their care. We believe our study results are generalizable to an international audience, but future research must be conducted in other countries with different health care and technology development systems.

This study does not come without limitations. First, we cannot completely eliminate the risk of introduction of bias to participants in this a priori study, where patient engagement is known to be beneficial in health research. To mitigate this bias, we used an open-ended focus group guide. Participants disagreed with one another, and questioned the need for patient engagement in AI in health research all together, making us comfortable that there was not strong social desirability bias or agreement bias in our cohort. To mitigate confirmation bias from our a priori literature review, we used multiple coders and acknowledged our role/goal of the study up front. Second, our patient participant sample was not representative of the general public’s educational background, which could have an impact on the understanding and perspectives on AI applications. Furthermore, our study sample did not contain any 1) self-identifying Indigenous people who may have unique views on AI and data in Canada, based on historical discrimination and colonialism, and they may further refine our proposed recommendations, or 2) people specifically identifying as caregivers, who are also under the definition of patient partner and have important perspectives for their loved one’s experiences. Given that the vast majority of participants were located within the Greater Toronto Area, future research should also seek out to engaging patient voices across the country and internationally.

In order to develop a holistic understanding of patient engagement practices in AI, we acknowledge the importance of incorporating the voices of an interdisciplinary group of participants, including health care workers and policy makers. Due to the ongoing pandemic and demands on health care workers at that time, their recruitment was not feasible and is an important area of future research. Despite this however, we believe the incorporation of interdisciplinary voices can assist in the further adaptations of our current guidelines on patient engagement in AI.

## Conclusion

AI in health care is a field that will continue to see rapid developments and have long standing implications on health and the health care system. In order to ensure that innovation continues to meet the needs and address issues critical to patients, quality patient engagement is required. We hope that our research assists in starting a dialogue on effective, representative and inclusive patient engagement practices within the field of AI in health care so that it becomes the standard of innovation.

### Supplementary Information

**Additional file 1. **Participant AI Educational Module.

**Additional file 2. **Patient Focus Group Guide.

## Data Availability

The datasets used and analyzed during the current study are available from the corresponding author on reasonable request.

## Abbreviation

AI: Artificial intelligence
