# Supplementary material for: Exploring patient perspectives on how they can and should be engaged in the development of artificial intelligence (AI) applications in health care
Source: BMC Health Serv Res. 2023 Oct 26;23:1163. doi: 10.1186/s12913-023-10098-2 (PMC10605984; doi:10.1186/s12913-023-10098-2)
Supplement: Supplementary file 1 — Additional file 1. Participant AI Educational Module. [file 12913_2023_10098_MOESM1_ESM.docx]

**Additional File 1: Participant AI Educational Module**

**Welcome to the artificial intelligence educational module.**
 
**1. Introduction**
 
***What is the purpose of this module?***
You have received this module because you are participating in the research study: Exploring multiple perspectives on how patients can and should be involved in the development of guidelines for patient engagement in artificial intelligence (AI) health research. 
 
**You do not need to know anything about artificial intelligence (AI) to participate in this study.**
 
This module was created to provide you with background knowledge on AI prior to participating in the study. We aim to equip all participants with a baseline level of AI knowledge to encourage participation during our focus groups. You are not expected to be an expert on AI, and you will likely have further questions after reading this module. At the end of this module there will be a list of resources for further learning; however, this is optional for the purposes of this study. 
 
***This module will:***
• Define AI and describe computer programming methods that underlie AI technologies
• Broadly explore the applications of AI in medicine
• Describe ethical considerations when applying AI in the context of health care 
• Review the findings of our systematic review on AI and patient engagement
 
***Where does this information come from?*** 
A variety of sources, including academic literature and consultations with experts in health information technology and AI. References will be cited at the bottom of each page of this module. An abbreviated glossary of **bolded blue** terms is located at the end of each section of this module and a complete glossary of all the terms used can be found at the end of the module.
 
***How long will this module take to complete?***
This module will take most people about **30 minutes to read**.  You can take breaks while completing the module, and return to it at a later session for completion. There is a short questionnaire at the end that will take up to 5 minutes to fill out.
 
***How does the module work?***  
The online module is delivered using a secure survey platform. You have received a custom link that saves your progress so you can return to this module at your convenience.

**2. Patient Engagement**
 
***What is a patient engagement?***
**Patient engagement** is a term used to describe the collaborations between patients and caregivers with health care providers and provincial organizations in order to improve the quality of health care (1). Some examples of engagement methods include user panels, focus groups, interviews, surveys, and patient advisory boards.  
 
***Why is patient engagement important for the future of AI in health care?*** 
 Advances in AI are driving innovation in most industries, including health care. Currently, AI is not widely used in health care. Although it is expected that AI will change health care in the near future, exactly how and in what ways remains unclear. Many factors will interact in complex ways, including primary care provider roles, patient preferences, policies, political considerations, and commercial interests. What is most important for the success of any AI-based technology is that its function matches the needs of patients, providers, and health administrators. To achieve this, we must be intentional in engaging a wide range of voices and experiences. 
 
**For the purpose of this study, we are aiming to develop formal guidelines for patient engagement in artificial intelligence development.**


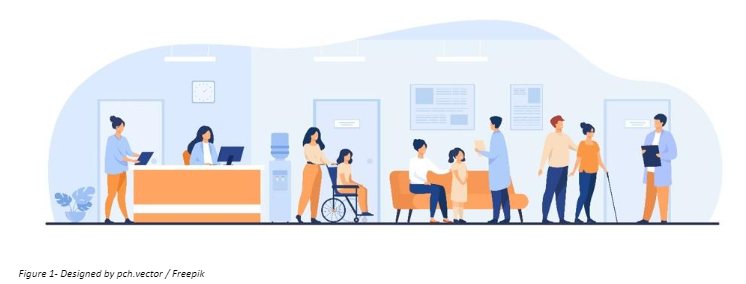

  
***Figure S1*** *(Designed by pch.vector / Freepik)*: Ontario’s Patient Engagement Framework,” n.d., 21.

Q3 **3. Artificial Intelligence: An Overview**
 
In this section, we will define artificial intelligence (AI) and review common computer programming methods used to create most everyday AI-based applications. By the end of this section, you should understand how deep learning methods fit within AI, and how AI relates to Big Data (Figure 2).


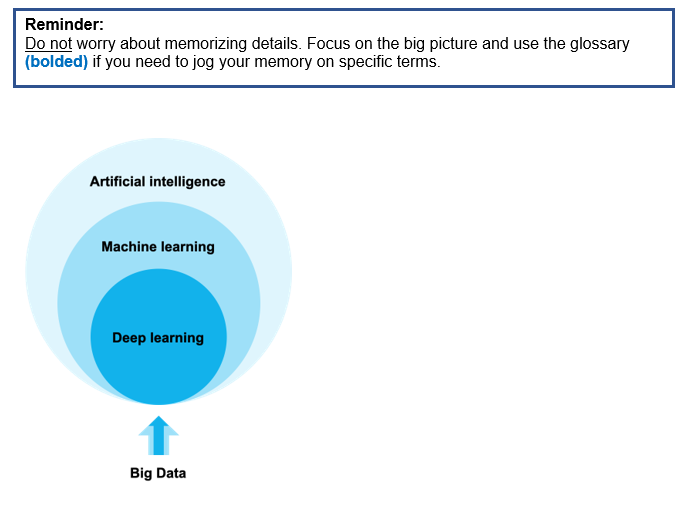


***Figure S2:*** *Artificial Intelligence, Machine Learning, and Big Data adapted by Jillian Macklin from* [*IBM: "Artificial Intelligence"*](https://www.ibm.com/cloud/learn/what-is-artificial-intelligence)

We will use the terms ‘algorithm’, ‘system’, ‘program’ and ‘method’ interchangeably when talking about AI. AI methods are computer algorithms. 
 
An **algorithm** is a set of step-by-step instructions for solving a problem. Algorithms can be simple or complex (2): 
 - An example of a simple algorithm: Instructing your phone to set a reminder for 9AM on Saturday
 - An example of complex algorithm: Identify pedestrians in an image
 
We will learn more below about how AI algorithms can be combined to produce applications with complex “intelligent” functions.

 (2) Matthew Hutson, “AI Glossary: Artificial Intelligence, in so Many Words,” Science 357, no. 6346 (2017), https://doi.org/10.1126/science.357.6346.19.

***What is artificial intelligence?***

**Artificial intelligence** is a broad field of science concerned with getting computers to do tasks that would normally require human intelligence. In this definition, intelligence can refer to processing information, reasoning and learning, planning actions, and communicating in natural language. Another way to think about AI is as a general-purpose prediction technology that estimates missing information from available information (3). 
 

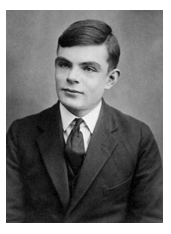

***Figure S3:***  [*Passport photo of Alan Turing, the father of artificial intelligence, at age 16*](https://commons.wikimedia.org/wiki/File:Alan_Turing_Aged_16.jpg#filelinks) *by unknown author /* [*CC BY 4.0*](https://creativecommons.org/licenses/by/4.0/)

 Alan Turing established the concept of AI in 1954 (4). Since then, computer scientists have developed a variety of methods that allow computers to mimic human intelligence. These methods can be grouped into several types; each type is suited to particular intelligent tasks. A combination of methods is usually used when developing AI technologies.
 
Today’s AI applications are "narrow." **Narrow AI** programs can only do what they were designed to do (5). "Narrow" AI applications are often better than humans at the tasks they were designed for but cannot develop additional skills without being programmed by humans. 
 
In contrast, **general AI** refers to a single system that can learn in different situations and then apply broad knowledge to solve any kind of problem – like the human mind! (5) This is what many people think of when they hear "AI." There are currently no actual examples of general AI. This is not surprising when we consider the complexity of human learning and problem-solving.


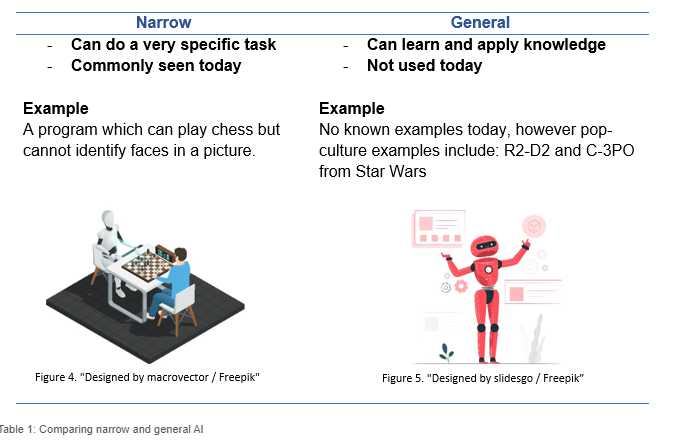


***Figure S4*** *(Designed by pch.vector / Freepik): Comparing narrow and general AI* 
     
**Abbreviated Glossary**
**Algorithm:** A set of step-by-step instructions for solving a problem. A computer algorithm can be simple (e.g. If it’s Saturday at 9 AM, send a reminder) or complex (e.g. Identify pedestrians).
**Artificial intelligence:** A broad field of science that is concerned with getting computers to do tasks that would normally require human intelligence; a-general purpose prediction technology that estimates missing information from prior information.
**Narrow AI:** AI programs that can only do what they were designed to do. "Narrow" AI applications are often better than humans at the tasks they were designed for but cannot develop additional skills without being programmed by humans.
**General AI:** A single system that can learn in different situations and then apply broad knowledge to solve any kind of problem. There are currently no actual examples of general AI.

  
(3) James Shaw et al., “Artificial Intelligence and the Implementation Challenge.,” Journal of Medical Internet Research 21, no. 7 (July 2019): e13659, https://doi.org/10.2196/13659.
(4) Bruce G Buchanan, “A (Very) Brief History of Artificial Intelligence,” AI Magazine 26, no. 4 (2005): 53, https://doi.org/10.1609/aimag.v26i4.1848.
(5) Leah Davidson, “Narrow vs. General AI: What’s Next for Artificial Intelligence?,” Springboard, 2019, https://www.springboard.com/blog/narrow-vs-general-ai/#:~:text=Narrow AI (ANI) is defined,and advances in that spectrum.”.

Q5 ***What is Big Data? How is it related to AI?*** 
 
**Big Data** is a term used to describe data produced by a variety of sources in large volumes at a fast pace. Big Data has grown across industries in the last three decades because of advances in computer processing power and storage, widespread adoption of mobile devices, and increased internet availability (6). AI methods allow us to condense and make sense of these datasets. These datasets can also be used to program a specific AI task. Advances in machine and deep learning in the last 10 years have depended on the availability of large, labelled “Big” datasets.
 
A **dataset** is a collection of data gathered using the same criteria for a specific purpose. Datasets from different sources can be shared and combined to create **linked datasets**. Linked datasets contain a broad range of information that can be analyzed to shed light on complex issues.

  
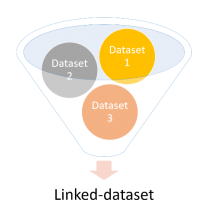

 ***Figure S5:*** *Depiction of linked-datasets* 
 
**An Example of Linked Datasets (7)**


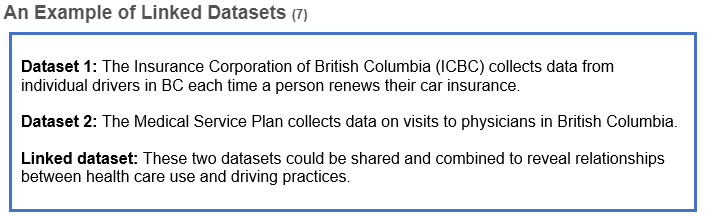


As information technology continues to develop, new sources of data will appear, allowing new linked datasets to be created. New linked datasets offer new opportunities for research in many areas, including AI.


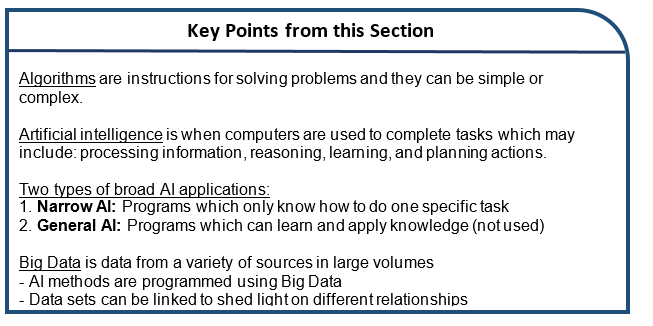


**Abbreviated Glossary**
 **Big Data:** The creation of large volumes of data at a fast pace
 **Dataset:**A collection of data gathered using the same criteria for a specific purpose
 **Linked dataset:**Dataset created when two datasets from different sources are combines. Linked datasets contain a broader range of information than either of the original datasets.
  
(6) A L Beam and I S Kohane, “Big Data and Machine Learning in Health Care,” Journal of the American Medical Association 319, no. 12 (2018): 1317–18, https://doi.org/10.1001/jama.2017.18391.
(7) Jack Teng et al., “Research Data Use in a Digital Society: A Deliberative Public Engagement” (Vancouver, British Columbia, Canada, 2019).
 
**How did you find understanding this section of the module?**

- Extremely easy (1)
- Somewhat easy (2)
- Neither easy nor difficult (3)
- Somewhat difficult (4)
- Extremely difficult (5)

The following sections describe some common AI methods that form the basis of most modern AI applications. All of these methods work best when applied to Big Data.
 
**3.1  Machine learning**
 
***What is machine learning?***
 
**Machine learning** refers to AI methods where a computer program learns from experience over time (8).

 
**An Example of Machine Learning: Spam Emails**

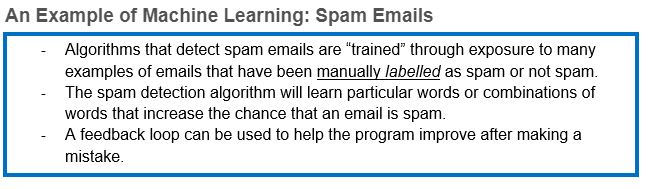

 
Spam detection is an example of **supervised learning**, in which labelled examples (in this case, emails labelled as spam or not spam) are included in a training dataset. 
 
In **unsupervised learning**, there are *no labelled examples* and an algorithm instead groups data by similarities. Unsupervised machine learning is increasingly used to drive discovery in basic science and medical research by revealing unexpected relationships among data (9). 


In both of types of learning, the performance of an algorithm and be tested by measuring the accuracy of the trained model on a dataset that was not used to train the model (commonly called a test dataset). 
 
As narrow AI, machine learning algorithms are usually more accurate than humans at the prediction tasks they are trained for. However, a machine learning AI system is not truly intelligent because it does not understand what it was trained to do. A spam detection algorithm can be great at filtering spam, but it will never understand what spam is and why it’s bad in the way humans do. If a new type of spam emerges for which the algorithm was not previously trained to recognize, it will likely have to be retrained by a (human) computer programmer to recognize it.

 **Machine learning forms the basis of most AI systems.**

 (8) Beam and Kohane, “Big Data and Machine Learning in Health Care.”
(9) Hutson, “AI Glossary: Artificial Intelligence, in so Many Words.”

**3.2 Deep learning**
 
***What is deep learning?***
 
**Deep learning** refers to a subtype of machine learning methods that identify hidden and complex patterns in a dataset. These patterns can be used to classify new data into a defined category (i.e. using supervised learning methods) or group new data by similarity (i.e. using unsupervised learning methods) with very high accuracy (10). 
 

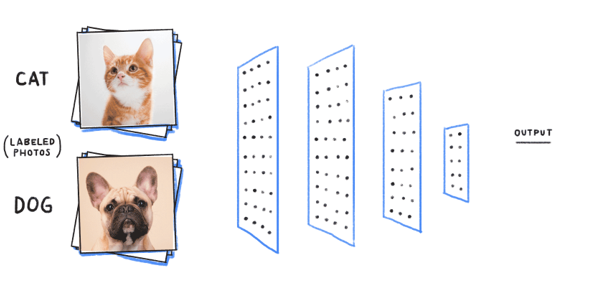

***Figure S6:*** [*Layers of a supervised deep learning algorithm*](https://miro.medium.com/max/3840/1*oB3S5yHHhvougJkPXuc8og.gif) *by unknown author*
  
Deep learning is a more recent type of AI that has improved existing technologies and helped create others that were not possible before, like self-driving cars. The distinguishing characteristic of a “deep” learning algorithm is the use of many layers (Figure 7). 
 
Each layer identifies different features of the data set; combining the layers allows for detection of complex patterns that can be used to make very accurate predictions. 
 
With “shallow” AI methods like classic machine learning, scientists must spend time: 
1) Identifying data features important for making accurate predictions, and 
2) Manually transforming these features into math a computer understands. 
 
Deep machine learning is powerful because it eliminates the need for manual feature selection and representation. This allows for much more complex prediction tasks.
 
Consider the example of distinguishing photos of cats from dogs – a popular project for AI developers-in-training (Figure 7). This initially seems like a simple task because we have been doing it all our lives – but think about what makes a dog visually different from a cat. Both have four legs, two eyes, and two ears - all roughly in the same position. Both have a tail and come in different colours. Dogs and cats can be similar in size.
 

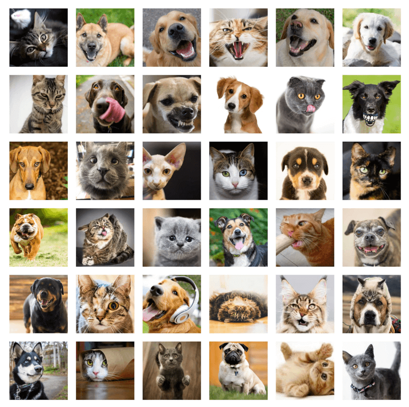

***Figure S7:*** *Example of photos of cats and dogs that can be used to train a deep learning image classification algorithm by unknown author*
 
So, how do we know they are not the same? Looking closely at the photos above, you will find no hard-and-fast rules for deciding. Yet, we can still tell the difference, but it's hard to pinpoint how we do it. Most of us will say that it has something to do with a subtle pattern of features that exist uniquely together. We have learned these patterns over time by exposure to examples, and probably by making a few mistakes. As you can imagine, these visual relationships are extremely difficult to represent in computer language.
 
Deep learning methods solve this problem by automatically detecting features of a dataset and defining relationships between them in mathematical terms a computer understands. This mimics the nuanced and highly accurate process of human pattern recognition. Humans can learn to categorize or group objects pretty well from a small number of examples. Deep learning algorithms learn from thousands or even millions of examples, making them extremely accurate at the prediction task they are designed for.
 
It is important to note that the “features” that a deep learning algorithm extracts from a dataset are abstract mathematical representations of data qualities. 
 
**They are not always concrete qualities that we can easily name.**
 
This can make it difficult to precisely understand how a deep learning AI system arrives at a prediction, even when it is correct. This is called **non-explainable AI**. For this reason, deep learning algorithms are often compared to a “black box.” (10)
 
Deep learning is well-suited to prediction problems in medicine because health and disease often involve complex interactions. As you can imagine, it is sometimes necessary for clinicians to understand contributing factors to an illness or injury to inform effective prevention and treatment. Efforts to develop “explainable” deep learning systems are ongoing.
 
 
(10) Yann Lecun, Yoshua Bengio, and Geoffrey Hinton, “Deep Learning,” Nature 521, no. 7553 (2015): 436–44, https://doi.org/10.1038/nature14539.

**3.3 Other AI methods**

 **Natural language processing (NLP)** refers to AI methods used to interpret human communication and reproduce it in various forms (9). In combination with deep learning, natural language processing is the basis of automated translation services like Google Translate, chatbots, and virtual personal assistants such as Apple’s Siri and Amazon’s Alexa. Most advanced NLP systems also use deep learning.
 
**Computer vision** refers to AI algorithms that interpret digital images or videos (10). Computer vision underlies facial recognition software and autonomous vehicles. Modern computer vision systems almost always use deep learning to perform their function. 
 

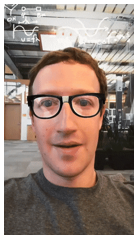

***Figure S8:*** [*Facebook CEO Mark Zuckerberg demonstrates Facebook’s facial filtering technology*](https://thumbs.gfycat.com/DearestHeavenlyCattle-size_restricted.gif) *by Mark Zuckerberg*  
 
For example, social media platforms like Instagram, Snapchat, Facebook, and TikTok use computer vision for their video filter technology. Filters use an AI algorithm to detect the features of a digital image that represent a human face. An animated filter is then applied to the face and can follow that face's movement in the frame.


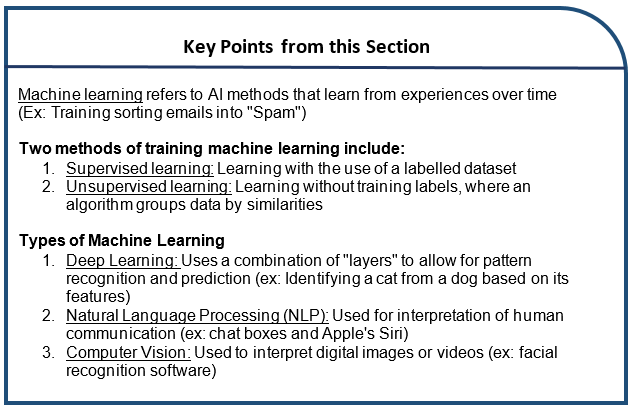

  


**Abbreviated Glossary**
 **Machine learning:**AI methods where a computer program learns from experience over time
 **Supervised learning:** A type of machine learning where an algorithm learns from labelled examples or positive and negative cases. Supervised learning is used to assign data into predetermined categories.
 **Unsupervised learning:**A type of machine learning where an algorithm learns without examples. Unsupervised learning is used to group data by similarities.
 **Deep learning:**A subgroup of machine learning algorithms that identify hidden patterns in a dataset. These patterns can be used either to classify new data into a predefined category (i.e. using supervised learning methods) or group new data by similarity
 **Non-explainable AI:**AI algorithms where the features of a dataset that contribute to a pattern are difficult or impossible to identify and define.
 **Natural language processing:** AI methods used to interpret and reproduce human communication
 **Computer vision:**AI algorithms that interpret digital images or videos
  
(9) Hutson, “AI Glossary: Artificial Intelligence, in so Many Words.”
(10) Yann Lecun, Yoshua Bengio, and Geoffrey Hinton, “Deep Learning,” Nature 521, no. 7553 (2015): 436–44, https://doi.org/10.1038/nature14539.

 **How did you find understanding this section of the module?**

- Extremely easy (1)
- Somewhat easy (2)
- Neither easy nor difficult (3)
- Somewhat difficult (4)
- Extremely difficult (5)

**If you would like to take a break and return to this module at a later time, this is a good time to do so.**

**4. AI in medicine and health care**
 
As we have learned, AI is a set of computer programming methods for analyzing large volumes of data from various sources to identify patterns. Patterns within data can be used to make highly accurate predictions. Predictions can add value to a wide range of tasks or problems in most industries, including health care. 
 
Several factors are driving the adoption of AI in health care today (11). First, labelled digital health datasets have grown exponentially in the last 30 years. 


The increase in labelled digital health datasets is the combined result of:
• Development of comprehensive **administrative datasets** that track a patient’s journey through a health care system
• Widespread adoption of **electronic medical records** (i.e. the computer software your doctor enters notes into during a visit)
• Advances in medical imaging technology
• Decreased costs of genomics technology (i.e. gene sequencing and mapping) resulting in more **genomic data**
• Broad uptake of “wearable” devices that track **physical data** like your heart rate, body mass index or your blood glucose levels

 These datasets can potentially be linked to provide a detailed data profile of an individual or a population. Additionally, during the mid-2000s, faster hardware was created to allow for the computational power necessary to analyze such large and high-variety datasets using deep learning.
 
(11) C David Naylor, “On the Prospects for a (Deep) Learning Health Care System,” JAMA 320, no. 11 (2018): 1099–1100, https://doi.org/10.1001/jama.

***What are the clinical applications of artificial intelligence?***
 
Clinical application of AI (especially deep learning) has been most rapid in medical disciplines that rely heavily on medical imaging. In many cases, deep learning AI systems outperform individual clinicians when interpreting medical images.

 For example, a group of AI scientists at Stanford University in California developed an AI algorithm to diagnose skin cancer from pictures of abnormal skin marks using deep learning and computer vision methods (Figure 10) (12). The algorithm was trained on 129,450 images of skin conditions labelled as cancer or not cancer by dermatologists. Algorithm performance correctly classifying skin cancer was compared to the performance of 21 dermatologists. Algorithm accuracy was equal to or better than that of most dermatologists involved in the study. To our knowledge, this tool has not yet been tested in clinical settings. 


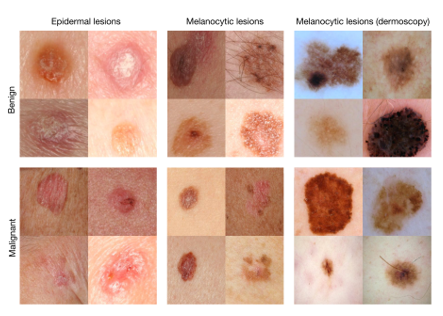
  
***Figure S9:****Example images from a test dataset used to assess the performance of a deep learning algorithm by Esteva et al.*
 
Increasingly, health care organizations are interested in applying deep learning AI to develop **risk stratification tools** that can organize patients according to their risk level for a specific health event (13). These tools can be used to **inform preventive care and support hospital operations** (e.g. staffing changes when care needs will likely be high).

 When implemented in clinical practice, a provider may increase support for individuals at increased risk for a particular event, shifting health care resources to those predicted by an algorithm to have the most need.
 
**An Example of Predictive Models: Predicting Hospital Readmission and Death (14)**

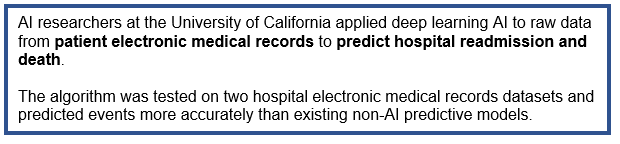

 
 
Other researchers have developed tools that can predict blood infection risk or delirium during a hospital stay, among many other examples.
 
It’s important to note that few algorithms developed by AI researchers have been widely implemented in clinical practice. This limits our understanding of how well prototype tools integrate within existing health care delivery systems or improve patient health.
 

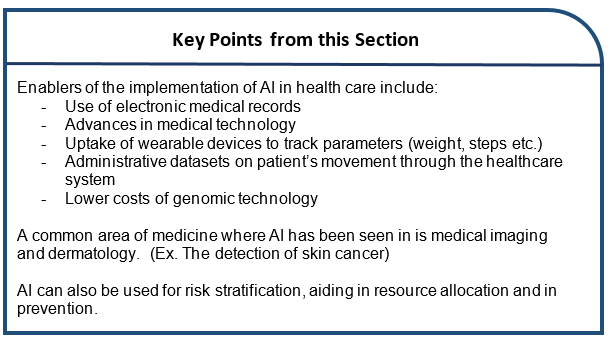


 **Abbreviated Glossary**
**Administrative data:**Data collected in the course of providing and/or paying for services (*e.g.* hospital admissions, physician visits)
**Electronic medical record:**Digital records kept by hospitals or individual providers that contain clinical data
**Genomic data:**Data describing DNA sequences
**Physical data:**Data about how your body is functioning from moment-to-moment, such as your heart rate of blood glucose levels; often collected from wearable devices
**Risk stratification:**Organizing people into risk levels that can be used to guide decisions.
  
(12) Esteva, Andre, Brett Kuprel, Roberto A. Novoa, Justin Ko, Susan M. Swetter, Helen M. Blau, and Sebastian Thrun. “Dermatologist-Level Classification of Skin Cancer with Deep Neural Networks.” Nature 542, no. 7639 (2017): 115–18. https://doi.org/10.1038/nature21056.
(13) Eric J. Topol, “High-Performance Medicine: The Convergence of Human and Artificial Intelligence,” Nature Medicine 25, no. 1 (2019): 44–56, https://doi.org/10.1038/s41591-018-0300-7.
(14) Alvin Rajkomar et al., “Scalable and Accurate Deep Learning for Electronic Health Records,” Npj Digital Medicine, no. January (2018): 1–10, https://doi.org/10.1038/s41746-018-0029-1.

**How did you find understanding this section of the module?**

- Extremely easy (1)
- Somewhat easy (2)
- Neither easy nor difficult (3)
- Somewhat difficult (4)
- Extremely difficult (5)

**5. Ethical considerations when applying AI to health data** 
 
AI has great potential to improve health and health care, but as with all innovations, there are limitations. These limitations raise some important ethical questions. 
 
***What is bias? Why does it matter?***
Many researchers have shown that non-health-related AI can worsen existing social inequalities by duplicating or worsening race, gender and other biases (15). **Bias** describes a preference for one thing, idea, person or group compared to another, usually in a way that is considered unfair. 
 
It is important to realize that **AI algorithms are only as good as the data they are trained on**. Some groups of people with better access to health care may contribute to health datasets more than other groups. AI algorithms trained on datasets that poorly represent certain groups may make less accurate predictions for members of those groups.
 
When used in health care decision making, biased predictions can worsen health inequality between groups.

 ***Consider the following hypothetical example (16):***
 

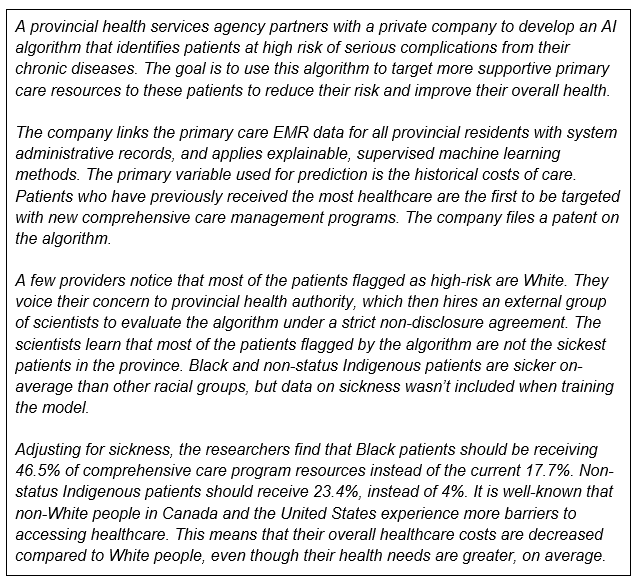


 This example is based on a published scientific analysis of a real AI algorithm applied to 200 million people each year in the United States (16). This algorithm is not a unique case but represents a general approach to risk prediction in the health sector. If health systems use biased algorithms when distributing health resources, the health of certain groups will improve while the health of others will get worse. 
 
**To combat bias, many researchers have emphasized the importance of including patients, clinicians, and ethicists from the beginning when developing AI applications for use in patient care.**
 
***What does that mean for AI and Patient Engagement?***
 We know that with the development of health interventions, when patients are consulted the intervention better suits the needs of the patient. The same reasoning can be applied to the development of AI and the need to emphasize the importance of the patient voice. 
 
In order to understand if and how patients have been consulted in the development of AI interventions in health care, we conducted a systematic review of the literature which is summarized in the next section.

 (15) Jenna Wiens et al., “Do No Harm: A Roadmap for Responsible Machine Learning for Health Care,” Nature Medicine 25, no. 9 (2019): 1337–40, https://doi.org/10.1038/s41591-019-0548-6.
(16) Ziad Obermeyer et al., “Dissecting Racial Bias in an Algorithm Used to Manage the Health of Populations,” Science 366 (2019): 447–53.


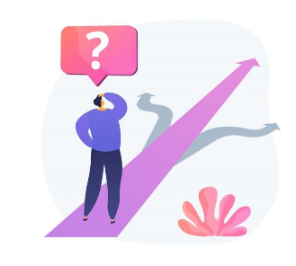

 ***Figure S10****: Designed by vectorjuice/Freepink*

 ***What other ethical considerations are there?***
 
Data privacy, data consent, and the public perception of data use is another area of uncertainty. Often, how the data is being collected and used is not transparent. Moreover, many private companies do not want to disclose their methods for developing a novel AI tool.
 
How can we trust that a tool marketed by a private company is unbiased and safe to use for all patients? Should developers and health care providers have to tell the public if they learn that an algorithm they are using is biased? What if a tool was instead developed and sold by researchers who published their methods in academic journals reviewed by other researchers?
 
And what about the **“black box”** quality of some deep learning AI systems? When is it important for the patient and provider to know what features of a dataset contribute to a prediction? Should we prioritize accuracy over interpretability?
 
As you can see, many important ethical questions arise when we think about the use of AI tools in health care settings.
 
**There are currently no clear answers to many of these questions.**


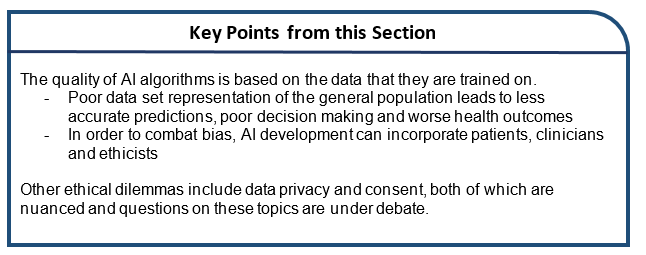

 
**Abbreviated Glossary**
**Bias:** A preference for one thing, idea, person or group compared to another, usually in a way that is considered unfair
 
 
**How did you find understanding this section of the module?**

- Extremely easy (1)
- Somewhat easy (2)
- Neither easy nor difficult (3)
- Somewhat difficult (4)
- Extremely difficult (5)

**6. Patient Engagement in AI: Summary of Systematic Review Findings**
 
***What is a systematic review?***
 A systematic review is a type of research method that involves extensively searching through the studies that have currently been published on a specific topic, within a specific time frame. It allows us to develop themes and ideas of what topics have and have not been researched within a given field. 
 
***What was our goal?***
 Our goal in conducting this systematic review was to determine if patients were engaged in AI development, and in what ways. We were interested in the methods and tools of engagement and how engagement impacted overall development of interventions. 
 
***How did we conduct the systematic review?***
We scanned all studies published between January 1st, 2000 to March 3rd, 2020. We searched the following databases in addition to hand-searching: MEDLINE, Medline Epub Ahead of Print & In-Process, Embase, Web of Science (conference proceedings), and CINAHL. 
 
***What key terms were used?*** 
We used both subject headings and text-word terms for Artificial Intelligence AND Medical Specialties or Health care Delivery AND Research, Development or Participation.
 
The following is a simplified overview of our two step inclusion/exclusion process:

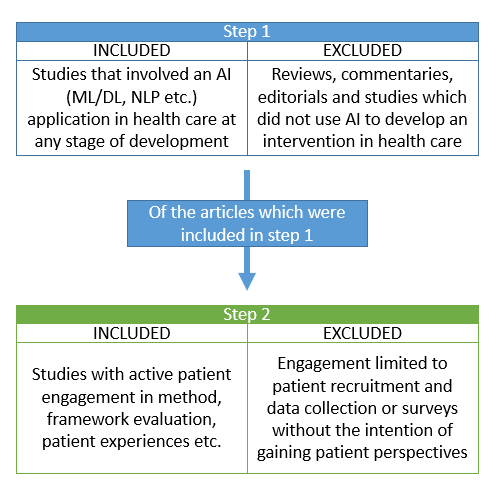

  
 ***Figure S11:*** *Stepwise process of systematic review, with inclusion and exclusion criteria*


 ***What did we find?***
Of the 8697 unique citations found, 704 were identified as articles relating to AI health care application development. Of those 704 articles, **37 studies (5.6%) incorporated some form of patient engagement.** (See Figure 13)
 

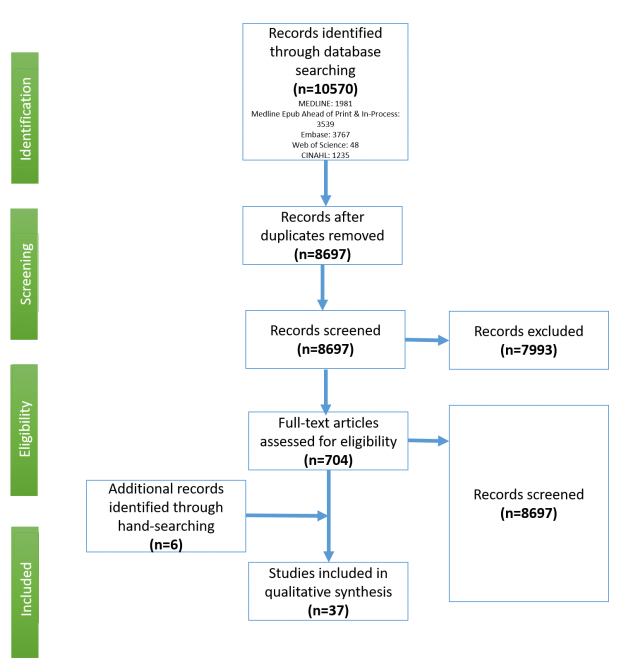


***Figure S12:*** *PRISMA diagram; Indicating the process and flow of the systematic review*
 
Of the studies that reported patient engagement:
 • The most common form of engagement was acceptability surveys, patient perspective interviews and workshop design activities 
 • The timing of engagement was often early in development in pilot stages
 
Common themes from these patient engagement sessions were a mixture of hope and skepticism of the implementation of AI. A main concern was the potential loss of human interaction and empathy in patient care. 

 **An Example of Patient Engagement in Research**
 Below is an example of a study included within our systematic review: 

 **Introducing artificial intelligence in acute psychiatric inpatient care: qualitative study of its use to conduct nursing observations.** (Barrera et al., 2020)

 The researchers of this article sought out to improve sleep quality and safety of acute mental health inpatients by utilizing a "digitally assisted nursing observations" through digital sensors, rather than having nurses enter patients rooms during the night. Here, the AI method is an intervention in the form of a "digitally assisted nursing observation".  
 
After the intervention was implemented, **they engaged patients by seeking feedback from former inpatients within the mental health ward**. Additionally, they surveyed front-line nursing staff to seek suggestions for improvement. Overall, they found that the new technology improved both patient and staff experiences during the night. (17)
  
***Our Recommendations for Future Patient Engagement***


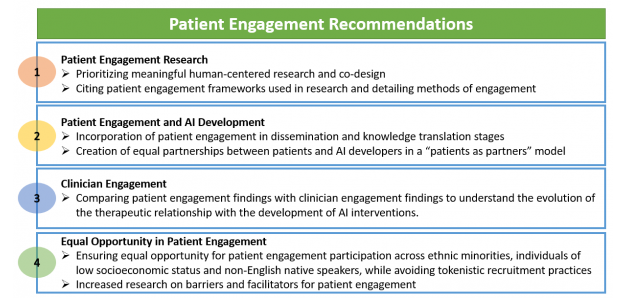

***Figure S13:*** *Summary of recommendations from systematic review findings*
 
From our systematic review, it is apparent that more rigorous evaluation and measurement of patient engagement impact is needed, in addition to understanding the barriers and facilitators of patient engagement in AI development. 
 
***Where do we go from here?***
As demonstrated by our systematic review, patients are often not included in the process of AI development of health care interventions. We know that when patient engagement is done well, that there are improvements in patient health in addition to decreases to health care costs (18-20). Building on this knowledge, we aim to hear your perspectives and experiences as a narrative in our focus groups to aid in the development of guidelines for developers to use on future patient engagement initiatives within AI. We look forward to hearing your unique perspectives during our focus group sessions. 
 
Please note that our systematic review is currently in the process of being updated to include papers published between March 3rd 2020 and April 15th, 2021 and updated findings will be shared once that process is completed. 
 
**If you would like a more detailed report of the search strategies and findings please email Samira Adus at samira.adus@mail.utoronto.ca**
  
(17) Barrera, A., Gee, C., Wood, A., Gibson, O., Bayley, D., & Geddes, J. (2020). Introducing artificial intelligence in acute psychiatric inpatient care: qualitative study of its use to conduct nursing observations. Evidence-Based Mental Health, 23(1), 34–38. https://doi.org/10.1136/ebmental-2019-300136
(18) Macklin JA, Djihanian N, Killackey T, MacIver J. Engaging Patients in Care (EPIC): A Framework for Heart Function and Heart Transplant–Specific Patient Engagement. CJC Open. 2019;1(2):43-46. doi:10.1016/j.cjco.2019.01.002 16.  
(19) Boivin A, Lehoux P, Lacombe R, Burgers J, Grol R. Involving patients in setting priorities for healthcare improvement: A cluster randomized trial. Implement Sci. 2014;9(1):1-10. doi:10.1186/1748-5908-9-24 17. 
(20) Shimmin C, Wittmeier KDM, Lavoie JG, Wicklund ED, Sibley KM. Moving towards a more inclusive patient and public involvement in health research paradigm: The incorporation of a trauma-informed intersectional analysis. BMC Health Serv Res. 2017;17(1):1-10. doi:10.1186/s12913-017-2463-1

**7. Summary of the Module**
  
Artificial intelligence is a broad field of science concerned with getting computers to do tasks that would normally require human intelligence. AI methods can be applied to large datasets to identify relationships and extract meaning. These relationships can be used to sort new data into predefined categories or group data by similarity. **Machine learning, deep learning, computer vision, and natural language processing** are all types of AI that can be used alone or together to get a computer to perform a narrowly defined task. All of these methods work best when applied to large datasets. 
 
Both growth in labelled digital health datasets and increases in computer processing power are driving the adoption of AI in health care today. Clinical application of AI has been most rapid in medical disciplines that rely most on medical imaging. There is growing interest in applying deep learning AI to develop **risk stratification tools** that can organize patients according to their risk level for a health event. To date, few algorithms developed by AI researchers or companies have been widely implemented in clinical practice. This limits our understanding of how well prototype tools integrate within existing health care delivery systems or improve health.
 
Electronic medical records are a rich source of data that can be linked to other datasets, including data about health system encounters and data from wearable devices. The AI methods we have discussed can be applied to these data to create a wide variety of applications that may add value to primary care patients and providers. It is not guaranteed that AI will transform primary care or any other areas of medicine. **Researchers studying this area have emphasized the importance of involving primary care patients, providers and health administrators in the process of developing useful AI applications that address the needs of end users.** It is also important to evaluate AI tools to be certain that they actually improve patient care. 
 
There are some limitations to AI that raise important ethical questions. **AI algorithms are only as good as the datasets they are trained on.** Datasets that poorly represent certain groups may result in AI algorithms that make less accurate predictions for members of those groups, leading to bias. When used in health care decision-making, biased predictions may worsen existing social inequalities in health. Issues like consent to data use are also important to consider when applying AI in health care settings.


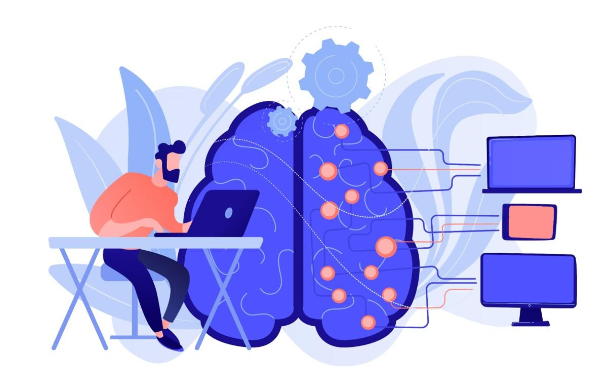
 
 ***Figure S14****: "Designed by vectorjuice / Freepik"*

Below are a list of additional resources for your own education on the topic of AI in health care. Please note that reviewing these resources is optional for the purpose of this study. 

 **8. Optional AI Educational Resources**
   
**News Articles**

 How can we keep algorithmic racism out of Canadian health care’s AI toolkit? By Hannah Alberga
 Link: <https://www.theglobeandmail.com/life/health-and-fitness/article-how-can-we-keep-algorithmic-racism-out-of-canadian-health-cares-ai/>

 Can AI tackle racial inequalities in healthcare? By Cody Goodwin
 Link: <https://www.bbc.com/news/technology-55940112>

 Can AI make health care more human? By Max Binks-Collier
 Link: <https://healthydebate.ca/2021/06/topic/ai-health-care-more-human/>

 AI tool ‘fundamentally changes the game’ for ICU care By Wendy Glauser
 Link: <https://healthydebate.ca/2021/06/topic/ai-tool-changes-the-game-icu-care/>

 Dealing with Bias in Artificial Intelligence By Craig S. Smith
 Link:[https://www.nytimes.com/2019/11/19/technology/artificial-intelligence-bias.html](http:// https://www.nytimes.com/2019/11/19/technology/artificial-intelligence-bias.html )


 **Videos and Documentaries**

 The AI Race: CBC’s The Passionate Eye
 Link: <https://gem.cbc.ca/media/the-passionate-eye/episode-158/38e815a-01284c9ff6a?cmp=passeye-pareto-docs>

 Coded Bias: On Netflix
 If you wish to learn about the contents of the documentary but do not have access to Netflix, this article summarizes the documentary:
 ‘Coded Bias’ is the most important film about AI you can watch today by Janus Rose
 Link: <https://www.vice.com/en/article/n7v8mx/coded-bias-netflix-documentary-ai-ethics-surveil>

 **Books**

 Deep Medicine: How Artificial Intelligence Can Make Healthcare Human Again. By Eric Topol

 Race After Technology. By Ruha Benjamin
  
 
**Journal Articles**

 Nadarzynski, T., Miles, O., Cowie, A., & Ridge, D. (2019). **Acceptability of artificial intelligence (AI)-led chatbot services in healthcare: A mixed-methods study. Digital Health**, 5, 2055207619871808–2055207619871808. <https://doi.org/10.1177/2055207619871808>
 Link: <https://journals.sagepub.com/doi/pdf/10.1177/2055207619871808>


 Bauer, G. R., & Lizotte, D. J. (2021). **Artificial Intelligence, Intersectionality, and the Future of Public Health.** American Journal of Public Health (1971), 111(1), 98–100. https://doi.org/10.2105/AJPH.2020.306006
 Link: <https://ajph.aphapublications.org/doi/10.2105/AJPH.2020.306006>


 Neri, E., Coppola, F., Miele, V., Bibbolino, C., & Grassi, R. (2020). **Artificial intelligence: Who is responsible for the diagnosis?** Radiologia Medica, 125(6), 517–521. https://doi.org/10.1007/s11547-020-01135-9
 Link: <https://link.springer.com/content/pdf/10.1007/s11547-020-01135-9.pdf>


 Amann, J., Blasimme, A., Vayena, E., Frey, D., & Madai, V. I. (2020). **Explainability for artificial intelligence in healthcare: a multidisciplinary perspective.** BMC Medical Informatics and Decision Making, 20(1), 310–310. https://doi.org/10.1186/s12911-020-01332-6
 Link: <https://bmcmedinformdecismak.biomedcentral.com/track/pdf/10.1186/s12911-020-01332-6.pdf>


 Chandler C, Foltz PW, Elvevåg B. **Using Machine Learning in Psychiatry: The Need to Establish a Framework That Nurtures Trustworthiness.** Schizophr Bull. 2020 Jan 4;46(1):11-14. doi: 10.1093/schbul/sbz105. PMID: 31901100; PMCID: PMC7145638.
 Link: <https://www.ncbi.nlm.nih.gov/pmc/articles/PMC7145638/> 


 Park, C. W., Seo, S. W., Kang, N., Ko, B., Choi, B. W., Park, C. M., Chang, D. K., Kim, H., Kim, H., Lee, H., Jang, J., Ye, J. C., Jeon, J. H., Seo, J. B., Kim, K. J., Jung, K. H., Kim, N., Paek, S., Shin, S. Y., … Yoon, H. J. (2020). **Artificial Intelligence in Health Care: Current Applications and Issues.** Journal of Korean Medical Science, 35(42), e379–e379. https://doi.org/10.3346/jkms.2020.35.e379
 Link: <https://jkms.org/Synapse/Data/PDFData/0063JKMS/jkms-35-e379.pdf>

 McCoy, L. G., Banja, J. D., Ghassemi, M., & Celi, L. A. (2020). **Ensuring machine learning for healthcare works for all.** BMJ Health & Care Informatics, 27(3). https://doi.org/10.1136/bmjhci-2020-100237
 Link: <https://informatics.bmj.com/content/bmjhci/27/3/e100237.full.pdf>


 Leiner, T., Bennink, E., Mol, C. P., Kuijf, H. J., & Veldhuis, W. B. (2021). **Bringing AI to the clinic: blueprint for a vendor-neutral AI deployment infrastructure.** Insights into Imaging, 12(1), 11–11. https://doi.org/10.1186/s13244-020-00931-1
 Link: <https://insightsimaging.springeropen.com/track/pdf/10.1186/s13244-020-00931-1.pdf>


 Scott, I. A. (2021). **Demystifying machine learning - a primer for physicians**. Internal Medicine Journal. https://doi.org/10.1111/imj.15200
 Link:<https://onlinelibrary.wiley.com/doi/abs/10.1111/imj.15200>

 Buruk, B., Ekmekci, P. E., & Arda, B. (2020). **A critical perspective on guidelines for responsible and trustworthy artificial intelligence.** Medicine, Health Care, and Philosophy, 23(3), 387–399. https://doi.org/10.1007/s11019-020-09948-1
 Link: <https://link.springer.com/article/10.1007/s11019-020-09948-1>


 Bertalan Meskó, & Marton Görög. (2020). **A short guide for medical professionals in the era of artificial intelligence.** NPJ Digital Medicine, 3(1), 126–126. https://doi.org/10.1038/s41746-020-00333-z
 Link: <https://www.nature.com/articles/s41746-020-00333-z.pdf>


 Paulus, J. K., & Kent, D. M. (2020). **Predictably unequal: understanding and addressing concerns that algorithmic clinical prediction may increase health disparities.** NPJ Digital Medicine, 3(1), 99–99. https://doi.org/10.1038/s41746-020-0304-9
 Link:  <https://www.nature.com/articles/s41746-020-0304-9.pdf>

**Glossary**
 
**Administrative data:** Data collected in the course of providing and/or paying for services (e.g. hospital admissions, physician visits)
 
**Algorithm:** A set of step-by-step instructions for solving a problem. A computer algorithm can be simple (e.g. If it’s Saturday at 9 AM, send a reminder) or complex (e.g. Identify pedestrians). 
 
**Artificial intelligence:** A broad field of science that is concerned with getting computers to do tasks that would normally require human intelligence; a-general purpose prediction technology that estimates missing information from prior information. 
 
**Bias:** A preference for one thing, idea, person or group compared to another, usually in a way that is considered unfair 
 
**Clinical data:** Data collected by a hospital or provider in order to provide appropriate healthcare services 
 
**Computer vision:** AI algorithms that interpret digital images or videos 
 
**Dataset:** A collection of data gathered using the same criteria for a specific purpose 
 
**De-identified data:** Data where identifiers such as a person’s name and address have been removed to reduce the possibility that the data could be traced back to any individual 
 
**Deep learning:** A subgroup of machine learning algorithms that identify hidden patterns in a dataset. These patterns can be used either to classify new data into a predefined category (i.e. using supervised learning methods) or group new data by similarity 
 
**Explainable AI:** AI algorithms where the features of a dataset that contribute to a pattern are possible to identify and define 
 
**General AI:** A single system that can learn in different situations and then apply broad knowledge to solve any kind of problem. There are currently no actual examples of general AI. 
 
**Genomic data:** Data describing DNA sequences 
 
**Linked dataset:** Dataset created when two datasets from different sources are combines. Linked datasets contain a broader range of information than either of the original datasets. 
 
**Machine learning:** AI methods where a computer program learns from experience over time 
 
**Narrow AI:** AI programs that can only do what they were designed to do. "Narrow" AI applications are often better than humans at the tasks they were designed for but cannot develop additional skills without being programmed by humans.
 
**Natural language processing:** AI methods used to interpret and reproduce human communication 
 
**Non-explainable AI:** AI algorithms where the features of a dataset that contribute to a pattern are difficult or impossible to identify and define 
 
**Physical data:** Data about how your body is functioning from moment-to-moment, such as your heart rate of blood glucose levels; often collected from wearable devices 
 
**Risk stratification:** Organizing people into risk levels that can be used to guide decisions. 
 
**Supervised learning:** A type of machine learning where an algorithm learns from labelled examples or positive and negative cases. Supervised learning is used to assign data into predetermined categories. 
 
**Unsupervised learning:** A type of machine learning where an algorithm learns without examples. Unsupervised learning is used to group data by similarities.

Thank you for completing this educational module.
