# Supplementary material for: Exploring patient perspectives on how they can and should be engaged in the development of artificial intelligence (AI) applications in health care
Source: BMC Health Serv Res. 2023 Oct 26;23:1163. doi: 10.1186/s12913-023-10098-2 (PMC10605984; doi:10.1186/s12913-023-10098-2)
Supplement: Supplementary file 2 — Additional file 2. Patient Focus Group Guide. [file 12913_2023_10098_MOESM2_ESM.docx]

**Additional File 2:** **Patient Focus Group Guide**

| **Preamble** |
| --- |
| Thank you all for agreeing to be interviewed. We are conducting this focus group in order to better understand how patients can and should be engaged in the implementation of artificial intelligence, known as AI, in health care interventions. Our goal in conducting these focus groups is to create formal guidelines for patient engagement in AI development. We appreciate the variety of experiences and expertise that you all have to enrich today’s discussion.  My name is Samira and I am a medical student at the University of Toronto in addition to the research coordinator for this project from the Upstream lab, which means that I will be conducting interviews and interpreting data.  During today’s session, I will be posing a series of open-ended questions to the group, with no right or wrong answer. My role is to guide the conversation in addition to listening to your experiences in order to learn from you all. Each of you will be compensated $30 for 1.5 hours of your time today.  I am also joined by Jillian, who is an MD/PhD student at the University of Toronto and Co-investigator for this study. She will help with any technical difficulties and privately message each of you to confirm your email and consenting to today’s session.  As a reminder, your participation is voluntary and you can choose to withdraw consent at any time by privately messaging Jillian. You may abstain from answering questions and please respect differences of opinion among the group. For privacy, please do not discuss the contents of today’s session outside of this group. Once data has been analyzed, you will receive the final report of our findings.  ***Are there any questions at this time?***  Our agenda for today’s session will firstly be some brief introductions, followed by some definitions and preliminary findings from our systematic review on AI and patient engagement, then to our focus group questions. If you have any questions throughout the session, please feel free to privately message them to Jillian and if they need to be addressed to the entire group, I will address them. |

| **Introductions** |
| --- |
| First off with introductions, can everyone please change their name on Zoom to their first name and pronouns. I will then call on individuals to briefly state their name, if employed, what you do for work and why you have decided to join the focus group today. |

| **Participant Education** |
| --- |
| Now that everyone is acquainted, we can transition into some educational pieces about AI and patient engagement. I will first start with some definitions to ensure we are all on the same page:  **Working Definitions**  **Artificial Intelligence:** Is a term used to describe computer software which uses specified formulas or algorithms to process information for a specific output. In the educational module that was shared with you all before today’s session there were many examples of what AI can look like: differentiating pictures of cats from dogs, playing chess, and diagnosing skin cancer.  **Patient Engagement:** Health Quality Ontario defines patient engagement as patients, family members and other informal caregivers and health care professionals actively collaborating to improve healthcare quality. Here, the term ‘patient’ is used to describe folks who are cared for in hospitals, in homes, through the community or in long-term care in addition to their caregivers.  **Systematic Review Findings**  Everyone has been emailed an information sheet about a recent systematic review conducted by the Upstream Lab, which investigated the degree to which patients have been engaged in AI development and the tools and methods used in engagement over the past 20 years. Notably from these findings was the following:   - Of the 8687 studies in AI development within healthcare found, only 5.6% mentioned patient engagement. - Among the 5.6% of studies which demonstrated patient engagement, the most common method of engagement was through surveys on the use, satisfaction and acceptability of AI interventions. Focus groups and individual patient interviews were among the less common methods of patient engagement. - Patients were commonly recruited through convenience sampling within the clinics of researchers conducting the study or through self-referral. - Among these studies that collected demographic data, most found participants identified as White, medically-stable or had mild disease, and had an understanding of AI terminology. |

| **Interview Questions** *(and prompts)* |
| --- |
| A. Establishing a participant narrative |
| 1. What surprised you most about the previous research we found on AI and patient engagement? 2. Does anyone have experience working within AI development?  - If so, can you describe your experience?   *Prompt: How were you recruited, what did you enjoy about the process, what didn’t work well etc.)* |
| B. Framework development |
| 1. We know that our healthcare system can better suit the needs of patients when there is patient engagement. What does good quality patient engagement look like to you?   *Prompt: Where is patient engagement being implemented? (Primary vs specialist care, treatment/intervention development, clinic feedback etc.). What are the effects of good patient engagement?*  Now I would like to break down what you have all imagined picture of perfect patient engagement to be with some specific components:   1. First, at what stage of development of AI technologies should **patients** be consulted? Why?   *Prompt: Examples may include problem identification, data collection, product design, product revisions, testing and implementation*   1. What is the best method to engage **patients** in developing AI technologies ?   *Prompt: Examples of engagement methods include individual consultations, focus groups/patient panels, surveys, patients serving as collaborators on committees etc.*   1. What are some barriers to engaging **patients** in meaningful ways within patient engagement frameworks in AI?   How do we overcome these barriers?  *Prompts: What is the role of understanding of AI as a barrier to patient engagement? Examples include understanding of AI, time, financial incentive, accessibility, privacy concerns etc.*   1. How do we ensure equal opportunity for **patients** to engage in AI development?   *Prompt: How do we ensure we are inclusive to patients with diverse backgrounds?*  *Specifically, equal opportunity and access to patient engagement channels across race/ethnic, sexual and religious minorities, low socioeconomic status, non-English speakers, etc.)*   1. What tools/training would you as a **patient** need to actively participate in patient engagement?   *Prompt: Whose responsibility is it to provide these tools to patients?*  *Examples include training, educational resources; elaborate on what medium of educational resources*  vii) How should post-engagement results be shared with **patients**?  *Prompt: What method? (summary document, formalized meeting etc.) How soon after engagement?*  viii) At the end of these series of focus groups that we are conducting for this study, we are planning a large meeting in the fall with a variety of stakeholders, including patients, health care providers, ethicists, policy makers and developers. From this meeting, we hope to refine guidelines for patient engagement in AI. With that said:  How can we prepare patients to engage at this meeting?  How can we facilitate this meeting to ensure patients are engaging in the guideline development?  *Prompt:* *Should we provide patients with their own speaking time? How do we overcome power-dynamics within the varying stakeholder groups?* |
| C. Future Quality Assessment |
| 1. How will we know if we’ve engaged **patients** well?   *Prompt:* *What methods can we use to measure if we’ve engaged patients well?*  *What values do we need to fulfil in good patient engagement?*  *How will we continue to improve over the coming years-decades?*  *Whose opinion of successful engagement matters?*   1. How may good patient engagement change the acceptability of AI in healthcare in the future?   *Prompt: Will we see changes in trust of AI interventions? Will patients' needs from providers change with the arrival of AI interventions?*  iii) Are there any topics that you wanted to discuss, but that have not been covered?  *Prompt: Anything that you initially imagined in your picture-perfect patient engagement that was not discussed in the session?* |

| **Conclusions** |
| --- |
| That marks the end of our focus group discussion today, thank you to everyone who has participated. As I’ve said, once all of the data from this study has been analyzed, we will be in contact via email with the results of the study during the late summer-early fall and with an opportunity to join the research team this fall in a collaborative session with patients, providers, health policymakers, and industrial developers.  Thank you again for your time, I am grateful for the discussion that we have had today, and please look to your email for your compensation. If you have any questions, please do not hesitate to reach out. |
